# High-density single nucleotide polymorphism markers analysis reveals the genetic diversity and population structure in tropical highland maize (Zea mays L.) inbred lines

**DOI:** 10.1371/journal.pone.0351845

**Published:** 2026-06-22

**Authors:** Worknesh Terefe Gebre, Demissew Abakemal Ababulgu, Tilahun Mekonnen Negassa, Tileye Feyissa Senbeta

**Affiliations:** 1 Holeta Agricultural Research Center, Ethiopian Institute of Agricultural Research, Holeta, Ethiopia; 2 Ambo Agricultural Research Center, Ethiopian Institute of Agricultural Research, Ambo, Ethiopia; 3 Biotechnology Research Center, Addis Ababa University, Addis Ababa, Ethiopia; Julius Kuhn-Institut, GERMANY

## Abstract

Genetic diversity is critical for crop improvement, germplasm conservation, and sustainable agriculture. It enables breeders to assess genetic relationships among germplasm, select suitable parents, and develop resilient varieties. In this study, a total of 11,203 single nucleotide polymorphism (SNP) markers were used to evaluate the genetic diversity of 93 maize inbred lines adapted to the East African tropical highlands. The results revealed moderate genetic diversity across the panel. Gene diversity, polymorphic information content (PIC), and genetic distance ranged from 0.10 to 0.67, 0.10 to 0.59, and 0.03 to 0.52, with mean values of 0.46, 0.40, and 0.44, respectively. Linkage disequilibrium (LD) analysis identified 36,904 SNP pairs (7.5% of 487,225 comparisons) showing relatively strong LD (*r*^2^ ≥ 0.20), with an overall mean *r*^2^ of 0.067. Genome-wide LD decayed to *r*^2^ = 0.2 at approximately 93.82 kb, suggesting rapid decay and substantial historical recombination. Analysis of molecular variance (AMOVA) revealed that 95% of the total variation resided within germplasm source groups, whereas 5% was attributed to differences among groups, indicating low to moderate genetic differentiation. Multivariate analyses, including neighbor-joining, principal component analysis, and population structure analysis, consistently grouped the lines into three clusters, which largely corresponded with pedigree information. The observed diversity highlights the presence of valuable alleles that can be harnessed in maize breeding to enhance productivity and resilience in highland environments. Furthermore, the identified SNP markers in this study provide a useful genomic resource for future studies, including marker-trait association studies aimed at identifying genomic regions underlying key agronomic traits and accelerate genetic improvement in challenging environments.

## Introduction

Maize (*Zea mays* L.) ranks as the third most important cereal crop globally, following wheat and rice, and is extensively cultivated for human consumption, livestock feed, and industrial purposes [1–3]. Global maize production increased from 313 million metric tons in 1971–1,162 million metric tons in 2020, reflecting its expanding role in global food systems. Currently, the leading producers include the United States, China, Brazil, the European Union, and Argentina [4].

In Sub-Saharan Africa (SSA), maize is the predominant cereal crop and serves as a primary caloric source for more than 300 million people, in addition to its use in livestock feed and as an industrial raw material [5–6]. Despite its significance, maize productivity in Ethiopia (4 t ha ⁻ ¹) remains considerably lower than the global average yield of 5.88 t ha ⁻ ¹ [7]. This low productivity is partly attributed to the narrow genetic base resulting from prolonged selection within locally adapted germplasm [8–9]. Limited genetic diversity restricts breeding progress and reduces the potential for developing high-yielding, climate-resilient cultivars. Rapid population growth and increasing food demand further emphasize the need to exploit existing genetic variation for maize improvement.

Maize germplasm is broadly classified into temperate, subtropical, and tropical groups based on latitudinal and environmental adaptation [10]. Tropical maize generally exhibits higher allelic diversity than temperate germplasm [11–12], making it a valuable source of genetic resources for developing climate-resilient cultivars [13]. Within the tropical group, maize is further categorized into lowland, mid-altitude, and highland types. Highland maize is particularly notable for its superior performance under low-temperature conditions where other adaptation groups perform poorly [14]. Tropical germplasm constitutes a major reservoir of genetic diversity [15–16], and exploiting this diversity is essential for breeding programs targeting productivity, stress tolerance, and climate change adaptation.

Understanding genetic diversity and population structure is fundamental for effective crop improvement. Knowledge of genetic diversity enables breeders to identify divergent parental lines, maximize heterosis, and develop hybrids with enhanced resilience to environmental stresses [17–18]. Analysis of population structure further enables the differentiation of breeding populations, the introgression of favorable alleles, and classification of inbred lines into heterotic groups, which is an essential step in hybrid maize development [19–21]. Collectively, these insights support effective parent selection and long-term genetic gain in maize breeding programs.

Molecular markers, particularly single nucleotide polymorphisms (SNPs), are indispensable for assessing genetic diversity because of their abundance, genome-wide distribution, reproducibility, and suitability for high-throughput genotyping [22–23]. Advances in genotyping platforms, especially genotyping-by-sequencing (GBS), have greatly enhanced the capacity for genome-wide SNP discovery and enabled detailed evaluation of genetic relationships, population structure, and linkage disequilibrium [24–25]. These high-resolution tools provide a robust framework for characterizing germplasm diversity and accelerating maize improvement.

In East Africa, recent breeding efforts have focused on developing highland-adapted maize inbred lines with improved productivity and resilience to cold stress, drought, and emerging diseases. Although Ethiopia possesses diverse maize germplasm, including unique highland-adapted types, few studies have employed high-density SNP markers to assess the genetic diversity of locally adapted inbred lines [26–27]. Earlier studies that relied on phenotypic traits or low-density markers provided limited resolution, leaving the genetic structure, subgroup classifications, and potential heterotic patterns largely unresolved.

Addressing this knowledge gap is crucial for strengthening national breeding programs. Detailed characterization of genetic diversity enables the identification of complementary parents for hybrid development, minimizes redundancy in breeding materials, and improves selection efficiency. Insights into population structure also inform association mapping and genomic-assisted breeding strategies. In highland environments, where low temperatures and multiple stresses limit maize performance, well-characterized and genetically diverse inbred lines are essential for accelerating hybrid development. We hypothesize that tropical highland-adapted maize inbred lines developed for East Africa possess substantial genetic diversity and exhibit a structured population pattern reflecting their diverse origins and breeding histories. Furthermore, we hypothesize that linkage disequilibrium decays relatively rapidly across the genome, indicating that these lines are suitable for high-resolution genomic analyses.

This study evaluated the genetic diversity and population structure of tropical highland-adapted maize inbred lines developed for East Africa using genome-wide SNP markers. Specifically, the study aimed to estimate key diversity parameters, examine population structure and subgroup formation, and elucidate genetic relationships among lines to support parent selection and genomic-assisted breeding in highland maize improvement.

## Materials and methods

### Plant materials

A total of 93 maize inbred lines with diverse genetic backgrounds were evaluated in this study. These lines were developed through hybridization followed by successive selfing using a pedigree breeding approach at the Ambo Agricultural Research Center (AARC) of the Ethiopian Institute of Agricultural Research (S1 Table). Among them, 28 lines were derived from Ethiopian highland accessions, 26 from early-generation lines introduced from CIMMYT Mexico, and 33 from CIMMYT Zimbabwe germplasm. The lines were advanced through repeated selfing to achieve homozygosity, reaching approximately S4 to S5 generations. In addition, six inbred lines representing parental lines of released varieties were obtained from AARC as locally adapted breeding materials. The four germplasm source groups were predefined based on origin and breeding history.

All lines were evaluated under optimum growing conditions, and selection was conducted across generations to retain genotypes with high grain yield, desirable agronomic performance, and early- to intermediate maturity. The inbred lines were also screened under field conditions for resistance to major diseases prevalent in tropical highland environments, including turcicum leaf blight, common rust, and gray leaf spot, and selected tolerant genotypes were advanced to subsequent generations.

### DNA extraction and SNP genotyping

Genomic DNA was extracted from fresh leaf tissues of three-week-old seedlings grown under greenhouse conditions at Melkasa Agricultural Research Center. Samples were collected in 96**-**deep-well plates, freeze-dried, and extracted using the NucleoMag Plant Genomic DNA Extraction Kit (Macherey-Nagel GmbH & Co. KG, Düren, Germany) following the DArT protocol [28]. DNA quality and concentration were assessed using a NanoDrop^TM^ 2000 Spectrophotometer (Thermo Scientific^TM^, USA) and 0.8% agarose gel electrophoresis.

Genotyping was performed using the genotyping-by-sequencing (GBS) method as described by Elshire, Glaubitz [29]. Genomic DNA was digested with ApeKI, barcoded adapters were ligated, and fragments were PCR**-**amplified. The libraries were sequenced as 77-bp single-end reads on an Illumina HiSeq 2500 platform (Illumina, San Diego, CA, USA).

### SNP data filtering and genetic analysis

Raw SNP data were filtered to exclude markers with minor allele frequency (MAF) < 0.05, heterozygosity > 0.02 [30], and missing data > 30% [6]. These thresholds ensured the retention of informative, high-quality markers by removing loci with low allelic variation, excess heterozygosity indicative of genotyping errors in inbred lines, and excessive missing data that could bias downstream analyses [6]. A moderate missing data threshold was used to balance marker retention and genome coverage, as stricter filtering substantially reduced SNP density in the GBS dataset. SNPs lacking chromosomal position information (13% of the total markers) were excluded from linkage disequilibrium (LD) analysis because physical position information is required for LD estimation. All relevant data are publicly available in the Figshare Repository at https://doi.org/10.6084/m9.figshare.32396424. Genetic diversity parameters, including MAF, gene diversity(GD) expected heterozygosity (He), and polymorphic information content (PIC) were calculated using PowerMarker v3.2.5 [31]. Genetic distances were estimated using Nei’s method [32], and a neighbor-joining (NJ) phylogenetic tree was constructed and visualized in MEGA v11 [33].

Pairwise linkage disequilibrium (LD) was estimated using the squared allele frequency correlation coefficient (*r*^2^) between SNP marker pairs within each chromosome in TASSEL v5.2.8 [34]. The default LD window size of 50 markers was used; therefore, LD was calculated between each SNP and its 50 adjacent markers. SNPs lacking chromosomal position information were excluded prior to analysis. Pairwise *r*^2^ values were plotted against physical distance (kb), and LD decay was assessed using locally weighted scatterplot smoothing (LOESS) in R [35] with 10-kb distance bins. LD decay trends were further modeled following the nonlinear expectation described by Hill and Weir (1988) [35]. The LD decay distance was defined as the physical distance at which *r*^2^ declined to 0.2. Analysis of molecular variance (AMOVA) was conducted in GenAlEx v6.5 [36] following the methods described by Excoffier and Smouse [37]. Principal component analysis (PCA) was performed using the *prcomp()* function in R v 4.4.1 [38], and visualized using *ggplot2* [39].

Population structure was inferred using STRUCTURE v2.3.4 [40] under an admixture model with correlated allele frequencies. The analysis was performed with a burn-in period of 10,000 iterations followed by 50,000 Markov chain Monte Carlo (MCMC) repetitions for K = 1–10. The optimum number of clusters (K) was determined using the Evanno method [41] implemented in STRUCTURE HARVESTER [42]. Inbred lines with membership probabilities (Q) ≥ 0.6 were assigned to a specific cluster, whereas those with Q < 0.6 were classified as admixed. This threshold was selected to account for residual heterogeneity and admixture commonly observed in diverse maize inbred panels.

## Results

### DArTseq marker characteristics and distribution

**Fig 1:**
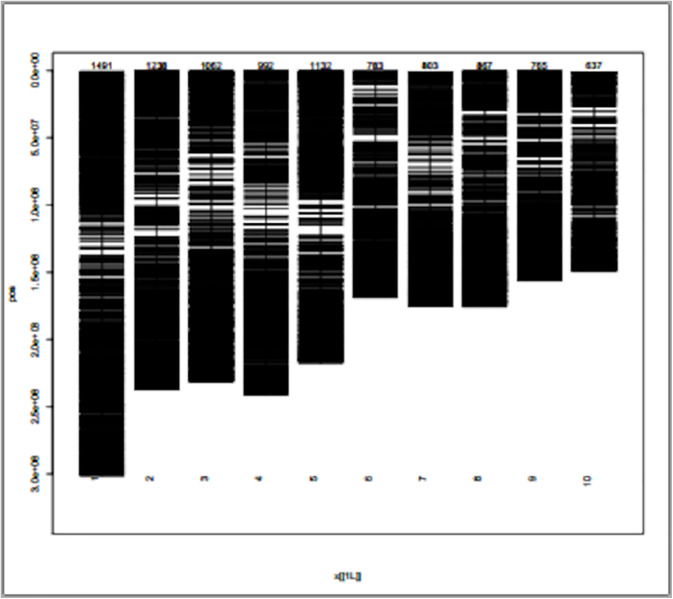
SNP distribution across the ten chromosomes of maize inbred lines.

Assessing genetic diversity is a fundamental in plant breeding because it provides a basis for developing high-yielding, stable, and stress-tolerant genotypes that contribute to food and nutritional security. Genotyping of the 93 maize inbred lines using the DArTseq platform initially generated 31,316 SNP markers. After quality control (QC) filtering, 11,203 high-quality SNP markers were retained, of which 9,770 were successfully aligned to the maize reference genome, whereas 1,433 mapped to unknown positions. The SNPs were distributed across all ten chromosomes, with chromosome 1 containing the highest number (1,491) and chromosome 10 the fewest (637). On an average, 977 SNPs were identified per chromosome (Fig 1).

### SNP Polymorphism and genetic diversity

**Fig 2:**
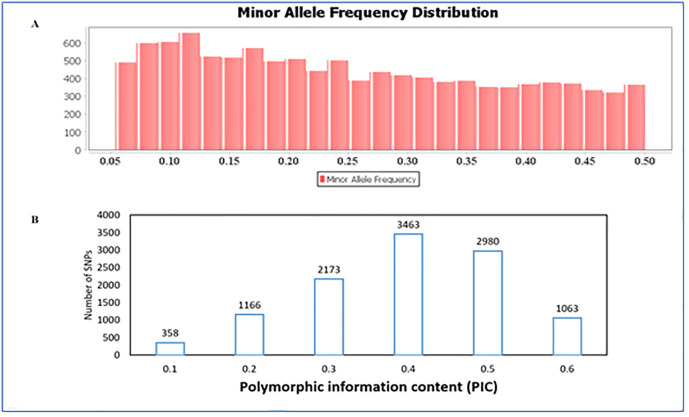
Frequency distribution of (A) minor allele frequency (MAF) and (B) polymorphic information content (PIC) of 11,203 DArTseq SNP markers.

**Fig 3:**
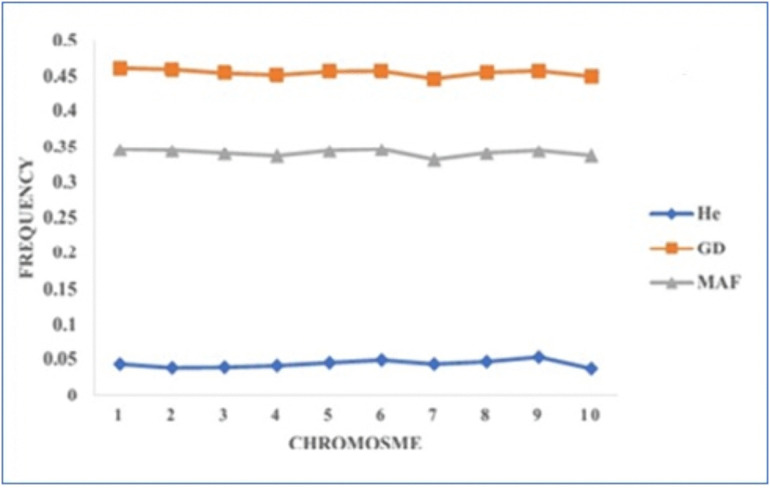
Distribution of summary statistics (MAF, He and GD) for the 11,203 SNPs across the ten chromosomes of all inbred lines.

Genome-wide diversity indices revealed considerable allelic variation across the maize inbred lines. Polymorphic information content (PIC) values ranged from 0.10 to 0.59, with a mean value of 0.40, indicating that the marker set was moderately to highly informative for assessing genetic diversity. Among the SNPs, 14% exhibited low PIC (< 0.3), 50% moderate (0.3–0.4), and 36% high (> 0.5) polymorphism (Fig 2B). Allele frequencies ranged from 0.05 to 0.66 (Table 1; Fig 2A). Expected heterozygosity (He), gene diversity (GD), PIC, and minor allele frequency (MAF) showed slight variation among chromosomes (Fig 3). Heterozygosity ranged from 0.01 to 0.70, with chromosomes 9 and 10 exhibiting the lowest values. Pairwise genetic distances among the inbred lines ranged from 0.03 to 0.52, with a mean of 0.44 (Table 1). The greatest genetic distance was observed between AML70 and AML2 (Additional S1 File), which were derived from different germplasm groups, whereas the smallest occurred between closely related sister lines AML31 and AML30.

**Table 1:** Level of polymorphism of 11,203 SNP markers in 93 maize inbred lines.

Statistic	MAF	He	GD	PIC	GDist
Minimum	0.05	0.01	0.10	0.10	0.03
Maximum	0.66	0.70	0.67	0.59	0.52
Mean	0.34	0.04	0.46	0.40	0.44

MAF = Minor allele frequency; He = expected heterozygosity; GD = Gene diversity; PIC = Polymorphic information content; GDist = Genetic distance.

### Allelic diversity within germplasm source groups

Genetic diversity indices revealed substantial variation within germplasm source groups but limited variation among groups (Table 2). Across all groups, the mean observed number of alleles (Na = 1.502), effective number of alleles (Ne = 1.205), expected heterozygosity (He = 0.153), and Shannon’s information index (I = 0.318) indicated moderate genetic diversity within the panel. Among the four predefined germplasm source groups, Group 1 exhibited the highest diversity with Na = 1.70, Ne = 1.313, He = 0.203, I = 0.318, and 70.33% polymorphic loci, suggesting a broader genetic base and greater allelic richness. Conversely, Group 4 showed comparatively lower diversity, with Na = 1.10, He = 0.097, and 22.66% polymorphic loci.

**Table 2:** Summary of genetic diversity statistics across loci for the four predefined germplasm source groups.

Germplasm source group	Na	Ne	Ho	He	I	(%P)
Group 1 (28)	1.70	1.31	0.00	0.20	0.32	70.33
Group 2 (26)	1.62	1.26	0.00	0.18	0.28	62.96
Group 3 (33)	1.56	1.20	0.00	0.14	0.23	59.16
Group 4 (6)	1.10	1.05	0.00	0.10	0.14	22.66
Overall	1.50	1.21	0.00	0.15	0.32	53.73

Na = Number of Alleles; Ne = effective number of alleles; Ho = Observed Heterozygosity; He = Expected Heterozygosity; I = Shannon’s Index; (%P) = Polymorphic loci

### Linkage disequilibrium (LD) analysis

Genome-wide linkage disequilibrium (LD) was estimated using pairwise *r*^2^ values across the ten chromosomes. A total of 487,225 marker pairs were analyzed, yielding a mean *r*^2^ value of 0.067. Among these, 36,904 pairs (7.57%) exhibited strong LD (*r*^2^ ≥ 0.2) (Table 3). Chromosome 1 contained the highest number of marker pairs (73,275), followed by chromosome 2 (61,900), whereas chromosome 10 contained the fewest (31,850). The highest chromosome-specific LD was observed on chromosome 9, with a mean *r*^2^ value of 0.075.

**Table 3:** Summary of linkage disequilibrium analysis among marker pairs.

Chromosome	TMNP	Mean *r*²	Mean marker distance (Mb)	Significant marker pairs (*r*² ≥ 0.2)
1	73,275	0.064	5.16	5,071 (6.92%)
2	61,900	0.074	4.92	5,668 (9.16%)
3	53,100	0.062	5.61	3,625 (6.83%)
4	49,600	0.058	6.27	2,914 (5.88%)
5	56,600	0.071	4.94	4,652 (8.23%)
6	39,150	0.068	5.47	3,027(7.73%)
7	40,150	0.06	5.61	2,513 (6.26%)
8	43,350	0.069	5.17	3,450 (7.94%)
9	38,250	0.075	5.21	3,486 (9.10%)
10	31,850	0.065	6.08	2,428 (7.60%)
Total	487,225	0.067	5.39	36,904 (7.57%)

Chromosome-wise summary of linkage disequilibrium (LD) statistics. TMNP = total marker pairs; mean *r*² = average LD between marker pairs; and mean marker distance (Mb) = average physical distance between SNP pairs. Significant LD was defined as *r*² ≥ 0.2. Genome-wide LD decay (*r*² = 0.2) was estimated separately from the LOESS-smoothed LD decay curve and occurred at approximately 93.82 kb.

**Fig 4:**
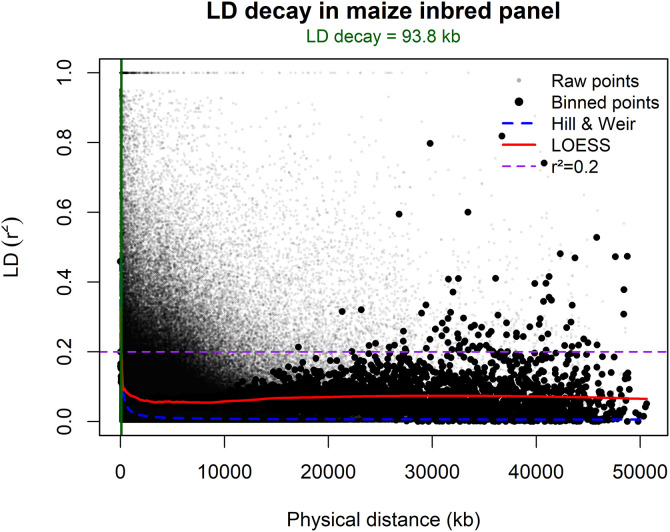
Linkage disequilibrium (LD) decay in the maize inbred panel. Pairwise LD (*r*^**2**^) is plotted against physical distance (kb). Gray points represent individual marker pairs, and black points indicate 10-kb binned averages. The red line shows the LOESS-smoothed trend, while the blue dashed line represents the nonlinear model of Hill and Weir. The horizontal dashed line marks the LD threshold (*r*^2^ = 0.2), and the vertical line indicates the estimated LD decay distance (~93.82kb).

Genome-wide LD decayed to *r*^2^ = 0.2 at approximately 93.82 kb based on the LOESS-smoothed curve (Fig 4). Average inter-marker distances ranged from 4.92 to 6.27 Mb across chromosomes, reflecting differences in marker distribution following SNP filtering.

### Analysis of molecular variance and genetic differentiation

Analysis of molecular variance (AMOVA) results is presented in Table 4, while the corresponding pairwise *F*_ST_ estimates are summarized in Table 5. AMOVA revealed that 95% of the total genetic variance was partitioned within germplasm source groups, whereas only 5% was attributed to variation among groups (Table 4). The overall *F*_ST_ value (0.05) indicated low to moderate genetic differentiation, suggesting weak but detectable genetic structure among the germplasm source groups. This pattern is supported by the low PhiPT value (0.01, *p* = 0.0001), which indicates limited genetic differentiation. The relatively high gene flow estimate (Nm = 4.35; S2 Table) further indicates substantial genetic exchange among groups, contributing to the predominance of within-group variation. These findings suggest extensive allele sharing and a high degree of common ancestry among the inbred lines. They are also consistent with population structure analysis, which identified three genetic clusters that do not strictly correspond to the four predefined germplasm source groups.

**Table 4:** Analysis of molecular variance (AMOVA) among the four germplasm source groups based on high-density DArTseq SNP markers.

Source	DF	SS	MS	Estimated of Variance components	% of Variation
Among groups	3	56187.16	18729.05	478.44	5
Within groups	89	741400.81	8330.35	8330.35	95
Total	92	797587.97		8808.78	100

PhiPT = 0.01; *p* = 0.0001. The *p*-value is based on 9,999 permutations; DF = degree of freedom; SS = sum of squares; MS = mean squares.

**Table 5:** Pairwise F_ST_ values (above diagonal) and pairwise genetic distances (below diagonal) among the four germplasm source groups.

Sub-populations	1	2	3	4
1	–	0.001	0.001	0.008
2	16440	–	0.001	0.009
3	16558	17328	–	0.007
4	17602	16769	17446	–
Overall *F*_ST_ value	0.05			
Overall *N*m value	4.35			

*N*m = number of migrants per generation; *F*_ST_ = Fixation index

Pairwise *F*_ST_ estimates among the germplasm source groups were uniformly low, ranging from 0.001 to 0.009 (Table 5), indicating minimal differentiation between most group pairs. Groups 1, 2, and 3 exhibited the least differentiation, whereas comparisons involving Group 4 showed relatively greater divergence, although overall differentiation remained weak. Patterns of genetic differentiation were further supported by genetic distance estimates, which indicated closer relationships among Groups 1, 2, and 3 and relatively modest divergence involving Group 4.

### Clustering and population structure

**Fig 5:**
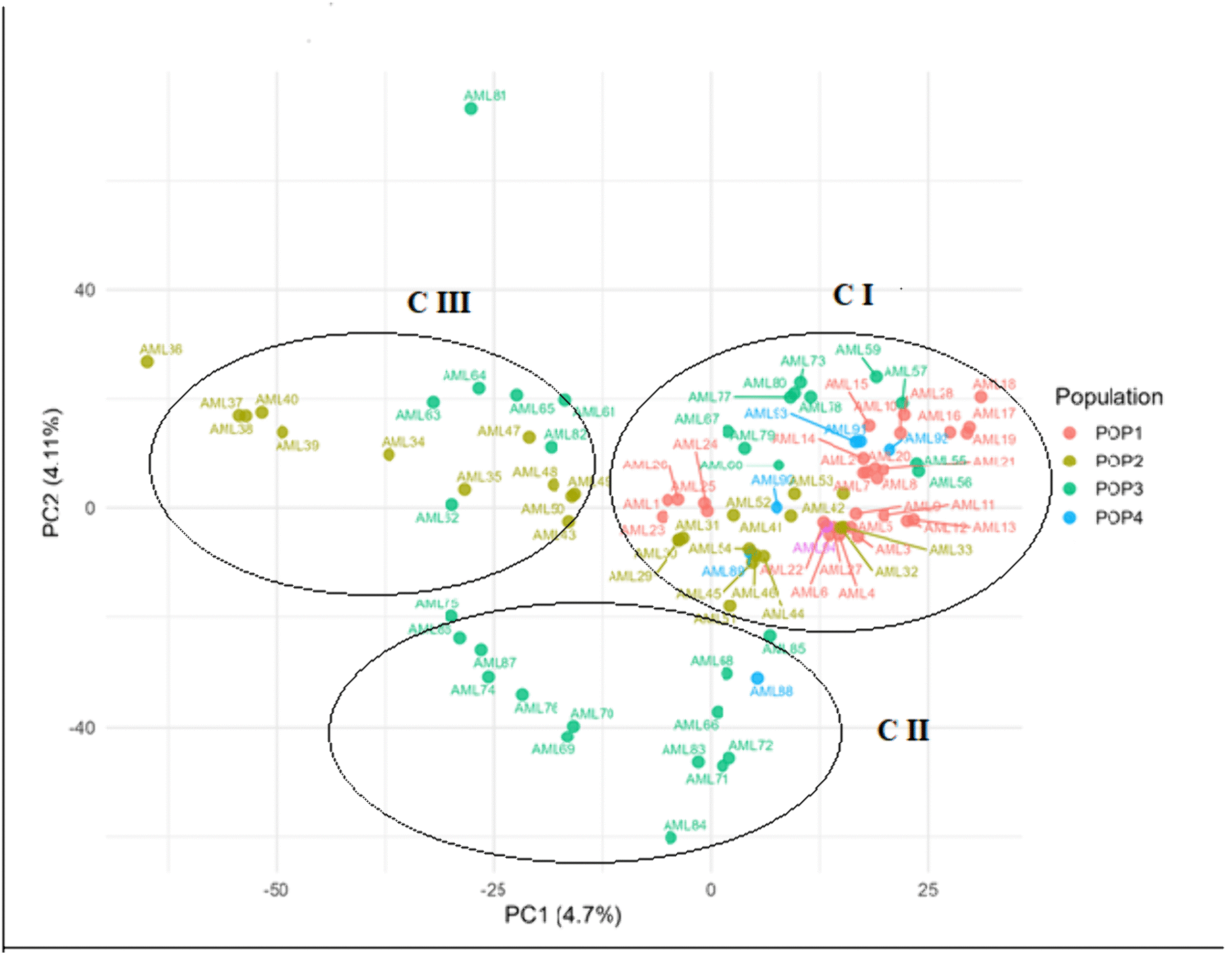
Principal component analysis based on 11,203 SNP markers grouped the four predefined germplasm source groups into three major clusters. Samples coded with the same color represent the same group.

Neighbor-joining (NJ), population structure, and principal component analysis (PCA) consistently revealed three genetic subgroups, reflecting distinct gene pools or evolutionary backgrounds. The NJ dendrogram grouped the 93 maize inbred lines into three main clusters (CI-CIII) (Fig 5). Cluster I included 40 inbred lines (43%), predominantly of exotic origin, although it also contained a few Ethiopian highland lines (AML20, AML27, AML94). Cluster II consisted of 13 lines (14%), representing a smaller group with relatively distinct genetic backgrounds. Cluster III included 38 lines (40.9%), mainly derived from the Kitale and F7215 testers, suggesting a more defined pedigree background. Two genotypes were identified as outliers, showing clear divergence from the main clusters. The clustering pattern generally corresponded with pedigree relationships, as closely related or sister lines grouped together, reflecting shared ancestry. For instance, lines from the AMB16N37-LD group clustered together, consistent with their derivation from testers such as F7215 (Kitale origin) and 142-1-e (Ecuador origin), highlighting the diverse genetic background of the germplasm.

**Fig 6:**
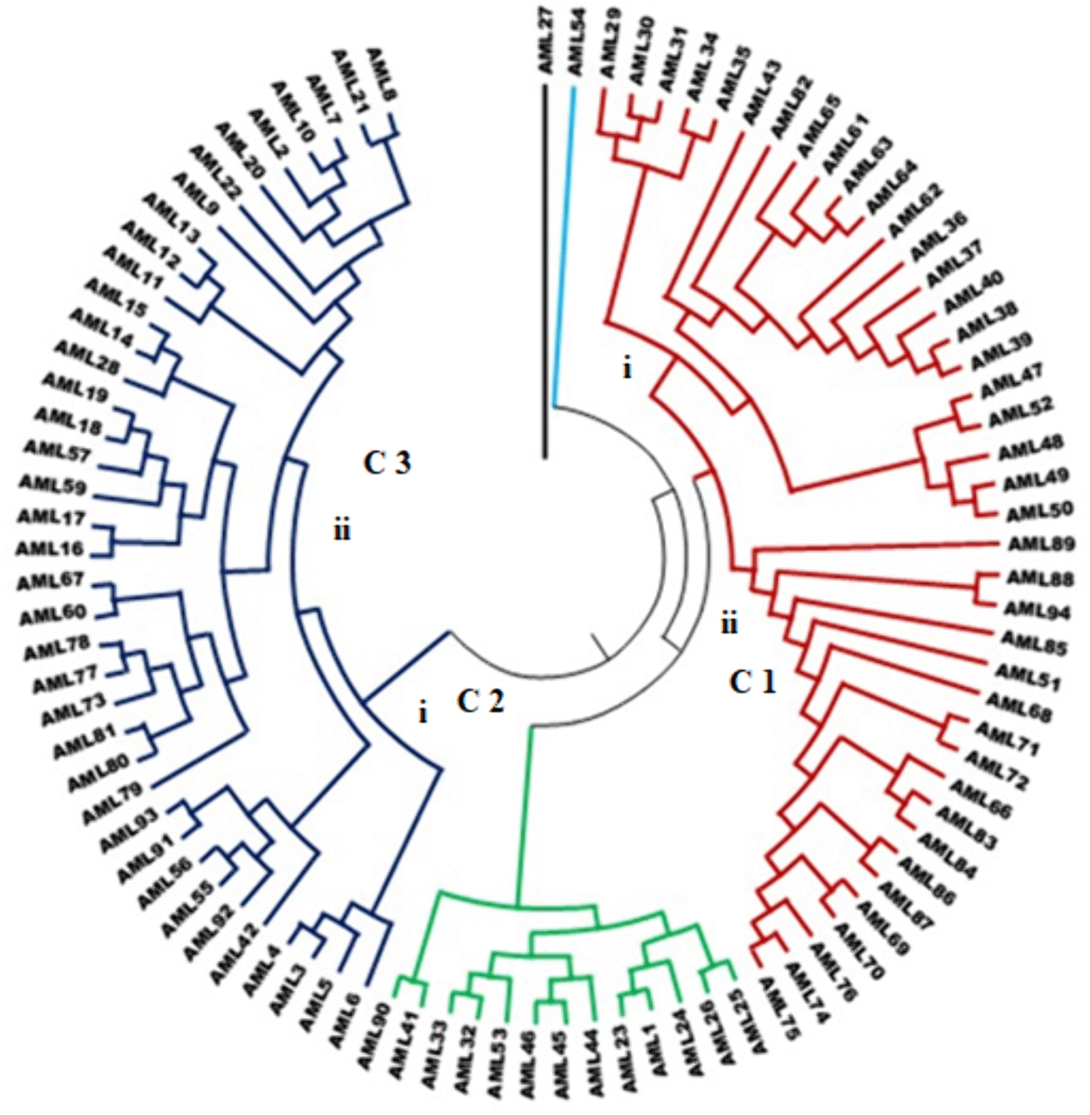
Neighbor-joining dendrogram showing the genetic relationships among 93 maize inbred lines based on SNP data, grouped into three clusters (C-I, C-II, and C-III), with two outlier genotypes.

Principal component analysis (PCA) also confirmed the presence of three genetic clusters (Fig 6). The first two principal components (PC1 and PC2) explained 8.81% of the total variation, contributing 4.7% and 4.11%, respectively. Lines from Group 1 were exclusively grouped in Cluster 1, whereas lines from the remaining groups were distributed across the remaining clusters, suggesting derivation from diverse parental crosses.

**Fig 7:**
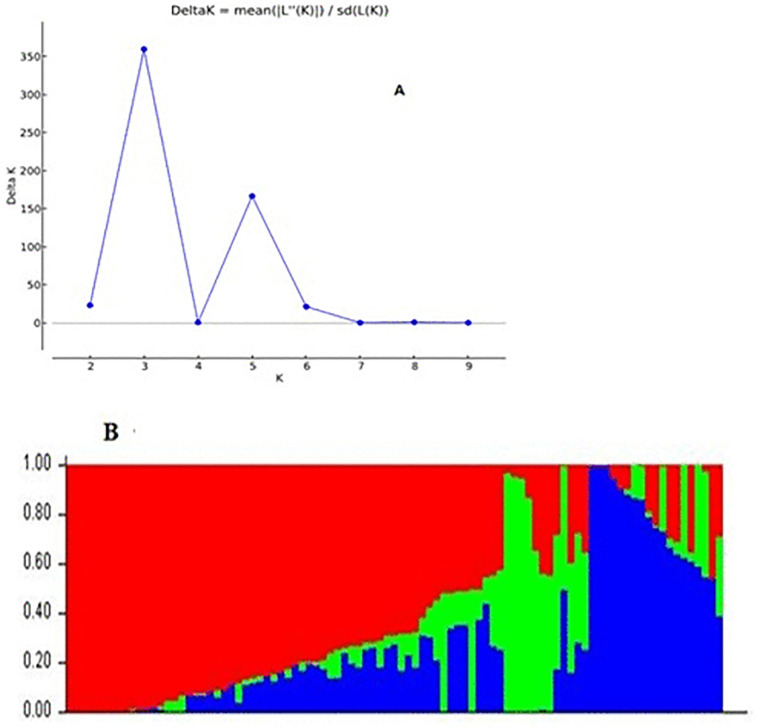
Population structure of 93 maize inbred lines inferred using STRUCTURE (K = 3). Each vertical bar represents an individual genotype, and colors indicate the proportion of membership (Q value) in each of the three inferred subpopulations. Genotypes were assigned to clusters using a threshold of Q ≥ 0.60, whereas those with lower values were considered admixed.

Population structure analysis using STRUCTURE supported the presence of three subpopulations, with a distinct peak in ΔK at K = 3 (Fig 7A, 7B). Based on a membership threshold (Q ≥ 0.60), 51 lines (54.8%) were assigned to subpopulation 1, five (5.4%) to subpopulation 2, and 15 (16.1%) to subpopulation 3 (S3 Table). The remaining 22 lines (23.7%) were classified as admixed. Clustering patterns were largely consistent across neighbor-joining, principal component analysis (PCA), and STRUCTURE analyses, although minor discrepancies in line assignment were observed, reflecting admixture and shared ancestry among groups.

## Discussion

Analysis of genetic diversity and population structure provides essential insights into the relationships, breeding potential, and adaptability of maize germplasm. In the present study, the observed genetic variation revealed moderate genetic diversity as indicated by gene diversity (He = 0.15) and polymorphic information content (PIC = 0.40). These findings suggest the presence of potentially valuable alleles that can be exploited in future breeding programs. In maize, such diversity is crucial for exploiting heterosis through hybrid development, which depends on crossing germplasm from genetically divergent clusters [43–45]. Therefore, identifying breeding materials carrying desirable alleles and associating these alleles with target traits is vital for efficient selection and hybrid development.

Although PIC values in this study were higher than those reported in previous studies [23,46,47], these differences are likely attributed to variation in SNP panels, allele frequency distributions, and filtering criteria. The relatively higher PIC values observed in the present study indicate a greater level of allelic diversity and marker informativeness within the evaluated germplasm. The moderate levels of gene diversity and genetic distance among lines further indicate the existence of considerable genetic variation, which is essential for achieving heterosis in hybrid combinations. Only two pairs of lines showed genetic distances below 0.05, suggesting limited redundancy among most of the inbred lines. The present findings are comparable with those reported by Ertiro [46], and Semagn [45]. Comparable levels of diversity were observed in Ethiopian highland maize accessions [27], supporting the uniqueness of the lines evaluated in this study.

Genetic diversity indices revealed pronounced variation within germplasm source groups but limited variation among groups (Table 3). The mean observed number of alleles (Na = 1.502), effective number of alleles (Ne = 1.205), expected heterozygosity (He = 0.153), and Shannon’s information index (I = 0.318) indicated moderate genetic diversity which is consistent with earlier studies in tropical maize [47–48]. Among the four germplasm source groups, Group 1, derived from Ethiopian highland accessions, exhibited relatively higher diversity, likely reflecting a broader genetic base and more balanced allele distribution. Such diversity makes this group a valuable source of alleles for breeding and hybrid development [49]. Conversely, Group 4 displayed comparatively lower diversity, possibly due to genetic bottlenecks, selection pressure, or restricted gene flow [50].

The relatively lower genetic diversity observed in Group 4 may reflect the combined effects of selection history and genetic drift. Recurrent selection during breeding can promote the fixation of favorable alleles, thereby reducing overall genetic variation, whereas genetic drift, particularly in smaller or closely related groups, can further diminish allelic diversity over successive generations. Overall, Group 1 represents a valuable source of genetic variation, while Group 4 may benefit from enrichment through introgression of diverse germplasm. These findings emphasize the importance of conserving genetically diverse germplasm source groups, particularly Group 1, to ensure sustained genetic gain and adaptability in maize improvement programs.

Linkage disequilibrium (LD) is a key determinant of mapping resolution and is influenced by recombination, selection, and population history. In this study, LD decayed to *r*^2^ = 0.2 at approximately 93.82 kb, indicating substantial historical recombination within the maize inbred panel. This estimate is consistent with previous reports in maize, where LD decay ranges from a few kilobases in highly diverse populations to several hundred kilobases in structured breeding populations depending on germplasm composition [52–53]. For example, Fan et al. [51] reported an average LD decay distance of 97.16 kb in newly released CIMMYT tropical maize inbred lines. Therefore, the decay distance observed in the present study falls within the expected range for tropical maize germplasm.

The LD pattern reflects both biological and methodological factors. As an outcrossing species with a high recombination rate, maize generally exhibits rapid LD decay, while population structure and relatedness among lines may contribute to localized LD persistence. In addition, LD estimates are influenced by marker density, allele frequency distribution, and sample size, as described by Hill and Weir [35]. The LD decay observed in this study suggests adequate mapping resolution for downstream genome-wide association studies (GWAS), consistent with previous reports [52], although higher marker density could further enhance genome coverage and improve the detection of trait-associated loci.

The analysis of molecular variance (AMOVA) revealed that 95% of the total genetic variation resided within germplasm source groups, whereas only 5% was attributed to differences among groups. The overall *F*_ST_ value (0.05) indicates low to moderate genetic differentiation, reflecting detectable genetic structure among germplasm source groups. This level of differentiation is typical for maize breeding materials, where extensive germplasm exchange and shared ancestry limit strong genetic separation [53–54].

The predominance of within-group variation suggests substantial genetic similarity among the maize inbred lines, likely resulting from recombination, mutation, and historical gene flow. Comparable patterns have been reported in previous studies, in which 97–98% of genetic variation occurred within maize inbred line groups by Ayesiga et al. [24]. The relatively high gene flow estimate observed in this study (Nm = 4.35) further supports limited differentiation and substantial gene exchange among groups, whereas lower Nm values reported in maize landraces [55–56] indicate more restricted gene flow. Despite the overall low to moderate differentiation, the observed genetic distances among groups particularly between Groups 1 and 4 may provide useful opportunities for exploring heterosis in maize breeding programs.

The relatively low variance explained by the first two principal components (~ 8.8%) indicates a complex and multidimensional genetic structure within the maize panel, highlighting the limitations of principal component analysis (PCA) when used alone. To better resolve this structure, complementary analyses using STRUCTURE and neighbor-joining (NJ) were performed. The consistency observed among PCA, NJ, and STRUCTURE results suggests a well-defined yet interconnected genetic architecture, reflecting the diverse ancestral origins and breeding histories of the inbred lines.

Although three genetic clusters were identified, the low *F*_ST_ and AMOVA estimates indicated relatively weak differentiation among groups, suggesting considerable shared ancestry and gene flow among subpopulations. The inferred population structure (K = 3) likely reflects contributions from multiple gene pools resulting from the integration of Ethiopian and exotic germplasm during inbred line development. The clustering of Ethiopian lines with introduced materials highlights substantial historical introgression and selection for adaptation to diverse agroecological conditions.

Although the burn-in period (10,000) and MCMC iterations (50,000) used in the STRUCTURE analysis were relatively modest, multiple independent runs yielded stable log-likelihood values [LnP(D)] and consistent ΔK support, indicating that the chosen parameters were sufficient for this dataset. However, higher iteration values may further improve the precision and stability of population structure inference, particularly in more complex or highly admixed populations.

Based on a membership coefficient threshold of Q ≥ 0.60, most of the 93 maize inbred lines were assigned to one of the three subpopulations, whereas a subset exhibited admixed ancestry (S3 Table; Fig 7). Subpopulation I contained the majority of lines, suggesting a shared genetic background likely shaped by common pedigree sources and selection for local adaptation. Subpopulation II comprised a smaller but clearly differentiated group, indicating a distinct breeding lineage. Subpopulation III included highly divergent lines which represent genetically distinct introduced germplasm.

The presence of admixed genotypes (Q < 0.60) reflects historical recombination and exchange among breeding materials, consistent with the mixed origin of the panel, including Ethiopian highland and CIMMYT-derived lines. This admixture pattern highlights the role of recurrent crossing and selection in combining desirable traits such as adaptation, yield potential, and stress tolerance. The observed genetic differentiation among subpopulations also suggests the existence of exploitable heterotic structure. Crosses between genetically distinct clusters, particularly those involving Subpopulation III are likely to maximize heterosis and improve hybrid performance, while admixed lines may serve as valuable intermediates for gene introgression and broadening the genetic base.

The observed admixture, supported by overlapping clusters and low differentiation indices (*F*_ST_ and AMOVA), reflects extensive gene flow and shared ancestry expected in breeding programs utilizing CIMMYT-derived materials. This genetic blending is advantageous because it broadens the allelic base, increases recombination potential, and reduces the risk of inbreeding depression. The 23.7% admixture rate aligns with previous reports [57–58] and underscores the dynamic nature of gene exchange in maize due to open pollination and the recurrent use of common parents.

From a breeding perspective, although genetic differentiation among clusters was low, the observed grouping provides a preliminary framework for parental selection. Crosses between lines from relatively distinct clusters, particularly between Group 1 and 4, offers opportunities for heterotic hybrid formation. The diverse ancestry represented in these groups can be harnessed to combine complementary alleles for yield potential, stress tolerance, and disease resistance consistent with the earlier reports [59–60]. Overall, the structured yet interconnected diversity observed in this study confirms that the evaluated maize inbred lines constitute a rich genetic reservoir for association mapping and marker-assisted selection, thereby contributing to the development of resilient, high-yielding cultivars adapted to diverse environments.

Although the present study focused on assessing molecular genetic diversity, the high-density SNP dataset generated provides a valuable foundation for future genomic analyses. In particular, these polymorphic markers could be utilized in genome-wide association studies (GWAS), when combined with phenotypic data to detect marker-trait associations for key agronomic traits such as grain yield, stress tolerance, and disease resistance. The moderate genetic diversity, substantial allelic variation, and detectable population structure also observed in this panel further support its suitability for association mapping and parental selection in breeding programs. Therefore, this study establishes an important genomic resource that can facilitate marker-assisted selection and genomic-assisted breeding aimed at improving maize adaptation and productivity in highland environments.

### Conclusion

This study revealed moderate to high genetic diversity among 93 tropical maize inbred lines based on high-density DArTseq SNP markers, as evidenced by an average PIC value of 0.40, relatively high gene diversity, and wide pairwise genetic distances among genotypes. The lines were grouped into three genetic clusters; however, genetic differentiation among predefined germplasm source groups was relatively low, as indicated by low *F*_ST_ values and predominance of variation within groups. Despite the low level of genetic differentiation, the observed diversity highlights the richness of the maize gene pool developed for tropical highland conditions. The observed genetic diversity highlights the potential of these inbred lines as valuable parental resources for maize improvement programs. Furthermore, the generated high-density SNP dataset also provides an important genomic resource for future applications, including GWAS, marker-assisted selection, and the efficient conservation and utilization of maize genetic resources adapted to highland agro-ecologies.

## Supporting information

S1 File Additional file: Nei’s genetic distance matrix among 93 maize inbred lines. (XLSX)

S1 Table List of maize inbred lines and their pedigree information used for genetic diversity. (DOCX)

S2 Table Pairwise number of migrants per generation (Nm) value among four germplasm source groups. (DOCX)

S3 Table Membership coefficients of 93 maize inbred lines inferred from population structure analysis (K = 3). Proportion of ancestry (Q values) of each genotype assigned to the three inferred sub-populations. Genotypes with Q ≥ 0.60 were assigned to a specific cluster, while those with Q < 0.60 were considered admixed. These data correspond to the population structure illustrated in Figure 7.
(DOCX)
