# Supplementary material for: High-density single nucleotide polymorphism markers analysis reveals the genetic diversity and population structure in tropical highland maize (Zea mays L.) inbred lines
Source: PLoS One. 2026 Jun 22;21(6):e0351845. doi: 10.1371/journal.pone.0351845 (PMC13286140; doi:10.1371/journal.pone.0351845)
Supplement: S1 Table — (DOCX) [file pone.0351845.s002.docx]

**Supporting Information Table S1**

Table S1. List of maize inbred lines and their pedigree information used for genetic diversity

| Entry | Name | Pedigree | Source | Initial founders |
| --- | --- | --- | --- | --- |
| 1 | AM**L**-1 | AMB01N216-2-2-1-3-3-#-#-B-B | EIAR | EIAR |
| 2 | AML-2 | AMB01N218-1-1-2-1-1-#-#-B-B | EIAR | EIAR |
| 3 | AML-3 | AMB01N219-2-2-1-2-1-#-#-B-B | EIAR | EIAR |
| 4 | AML-4 | AMB01N219-2-2-1-2-3-#-#-B-B | EIAR | EIAR |
| 5 | AML-5 | AMB01N219-2-2-2-1-1-#-#-B-B | EIAR | EIAR |
| 6 | AML-6 | AMB01N219-2-2-3-1-1-#-#-B-B | EIAR | EIAR |
| 7 | AML-7 | AMB01N2124-2-1-2-2-1-#-#-B-B | EIAR | EIAR |
| 8 | AML-8 | AMB01N2129-1-1-1-2-1-#-#-B-B | EIAR | EIAR |
| 9 | AML-9 | AMB01N2129-2-3-1-2-1-#-#-B-B | EIAR | EIAR |
| 10 | AML-10 | AMB01N2141-1-2-3-3-1-#-#-B-B | EIAR | EIAR |
| 11 | AML-11 | AMB01N2142-1-1-1-1-1-#-#-B-B | EIAR | EIAR |
| 12 | AML-12 | AMB01N2142-1-2-1-1-1-#-#-B-B | EIAR | EIAR |
| 13 | AML-13 | AMB01N2142-1-2-1-2-2-#-#-B-B | EIAR | EIAR |
| 14 | AML-14 | AMB01N2157-1-1-1-1-1-#-#-B-B | EIAR | EIAR |
| 15 | AML-15 | AMB01N2157-1-1-3-2-1-#-#-B-B | EIAR | EIAR |
| 16 | AML-16 | AMB01N2166-2-3-1-1-1-#-#-B-B | EIAR | EIAR |
| 17 | AML-17 | AMB01N2166-2-3-1-3-2-#-#-B-B | EIAR | EIAR |
| 18 | AML-18 | AMB01N2166-2-3-3-1-2-#-#-B-B | EIAR | EIAR |
| 19 | AML-19 | AMB01N2166-2-3-3-2-1-#-#-B-B | EIAR | EIAR |
| 20 | AML-20 | AMB01N2172-1-1-1-1-1-#-#-B-B | EIAR | EIAR |
| 21 | AML-21 | AMB01N2187-1-2-2-2-1-#-#-B-B | EIAR | EIAR |
| 22 | AML-22 | AMB01N2191-1-1-1-1-1-#-#-B-B | EIAR | EIAR |
| 23 | AML-23 | AMB01N21102-1-2-1-1-1-#-#-B-B | EIAR | EIAR |
| 24 | AML-24 | AMB01N21102-1-2-1-2-1-#-#-B-B | EIAR | EIAR |
| 25 | AML-25 | AMB01N21102-1-2-2-1-2-#-#-B-B | EIAR | EIAR |
| 26 | AML-26 | AMB01N21102-1-2-2-2-2-#-#-B-B | EIAR | EIAR |
| 27 | AML-27 | AMB01N21128-1-2-1-1-1-#-#-B-B | EIAR | EIAR |
| 28 | AML-28 | AMB01N21183-2-3-2-1-2-#-#-B-B | EIAR | EIAR |
| 29 | AML-29 | B.T.Z.T.R.L -83-B-1-3-1-2-2-3-#-#-#-#-B-B | EIAR | CIMMYT_MEXICO |
| 30 | AML-30 | B.T.Z.T.R.L -83-B-1-3-1-3-1-2-#-#-#-#-B-B | EIAR | CIMMYT_MEXICO |
| 31 | AML-31 | B.T.Z.T.R.L -83-B-1-3-1-3-1-3-#-#-#-#-B-B | EIAR | CIMMYT_MEXICO |
| 32 | AML-32 | B.T.Z.T.R.L -8-B-2-1-1-2-1-1-#-#-#-#-B-B | EIAR | CIMMYT_MEXICO |
| 33 | AML-33 | B.T.Z.T.R.L -8-B-2-1-1-3-1-3-#-#-#-#-B-B | EIAR | CIMMYT_MEXICO |
| 34 | AML-34 | B.T.Z.T.V.C -43-B -2-3 -1-1-1-1-#-#-#-#-B-B | EIAR | CIMMYT_MEXICO |
| 35 | AML-35 | B.T.Z.T.V.C -43-B -2-3 -1-3-2-#-#-#-#-#-B-B | EIAR | CIMMYT_MEXICO |
| 36 | AML-36 | B.T.Z.T.V.C -43-B -2-1 -2-2-2-#-#-#-#-#-B-B | EIAR | CIMMYT_MEXICO |
| 37 | AML-37 | B.T.Z.T.V.C -43-B -2-1 -3-1-1-3-#-#-#-#-B-B | EIAR | CIMMYT_MEXICO |
| 38 | AML-38 | B.T.Z.T.V.C -43-B-1-3 -1-3-2-#-#-#-#-B-B | EIAR | CIMMYT_MEXICO |
| 39 | AML-39 | B.T.Z.T.V.C -43-B-1-3 -1-1-2-#-#-#-#-B-B | EIAR | CIMMYT_MEXICO |
| 40 | AML-40 | B.T.Z.T.V.C -43-B-1-3 -1-3-2-#-#-#-#-#-B-B | EIAR | CIMMYT_MEXICO |
| 41 | AML-41 | B.T.Z.T.V.C -43-B-1-2 -1-1-1-#-#-#-#-#-B-B | EIAR | CIMMYT_MEXICO |
| 42 | AML-42 | B.T.Z.T.V.C -43-B-1-2 -1-2-1-#-#-#-#-#-B-B | EIAR | CIMMYT_MEXICO |
| 43 | AML-43 | B.I.Z.T.V.C -83-B-3-3-3-1-#-#-#-#-#-B-B | EIAR | CIMMYT_MEXICO |
| 44 | AML-44 | B.T.Z.T.V.C -138-B-2-3 -2-1-1-#-#-#-#-#-B-B | EIAR | CIMMYT_MEXICO |
| 45 | AML-45 | B.T.Z.T.V.C -138-B-2-3 -3-1-2-1-#-#-#-#-B-B | EIAR | CIMMYT_MEXICO |
| 46 | AML-46 | B.T.Z.T.V.C -171-1-1-2-1-2-1-#-#-#-#-#-B-B | EIAR | CIMMYT_MEXICO |
| 47 | AML-47 | B.T.Z.T.V.C -171-1-1-2-2-2-#-#-#-#-#-#-B-B | EIAR | CIMMYT_MEXICO |
| 48 | AML-48 | B.T.Z.T.V.C -171-1-1-2-3-2-#-#-#-#-#-#-B-B | EIAR | CIMMYT_MEXICO |
| 49 | AML-49 | B.T.Z.T.V.C -172-1-1-3-1-2-2-#-#-#-#-#-B-B | EIAR | CIMMYT_MEXICO |
| 50 | AML-50 | B.T.Z.T.V.C -172-1-1-3-2-1-2-2-#-#-#-#-B-B | EIAR | CIMMYT_MEXICO |
| 51 | AML-51 | B.T.Z.T.V.C -172-1-1-3-3-1-1-#-#-#-#-#-B-B | EIAR | CIMMYT_MEXICO |
| 52 | AML-52 | SINT TSR.B.T.Z.T.19P-1P-1P-2P-1P-5-1-1-3-#-#-#-#-B-B | EIAR | CIMMYT_MEXICO |
| 53 | AML-53 | B.T.Z.R.L.BA90-12-1-1P-1P-1-1-1-1P-3-1-1-#-#-#-#-#-B-B | EIAR | CIMMYT_MEXICO |
| 54 | AML-54 | B-62.5%9A TSR-19P-3P-1P-2P-1P-1P-1-1-1-3-#-#-#-#-B-B | EIAR | CIMMYT_MEXICO |
| 55 | AML-55 | [[KIT/SNSYN[N3/TUX]]c1F1-##(GLS=2)-32-2-1-2-1-2/L120]-1-1-1-B-1-B-B | EIAR | CIMMYT_ZIMBABWE |
| 56 | AML-56 | [[KIT/SNSYN[N3/TUX]]c1F1-##(GLS=2)-32-2-1-2-1-2/L120]-1-1-2-B-2-B-B-B | EIAR | CIMMYT_ZIMBABWE |
| 57 | AML-57 | [AMB01N2166-2-3-3-2/F7215]-3-2-1-B-1-B-B-B | EIAR | CIMMYT_ZIMBABWE |
| 59 | AML-59 | [AMB01N2166-2-3-3-2/F7215]-7-3-3-B-1-B-B-B | EIAR | CIMMYT_ZIMBABWE |
| 60 | AML-60 | [{HIDA 20 X CML 349)/G9AC7HS98-1)-1-1-1/F7215]-2-2-2-B-1-B-B-B | EIAR | CIMMYT_ZIMBABWE |
| 61 | AML-61 | [B.T.Z.T.V.C -43-B -2-2 -1-1-#/F7215]-4-1-2-B-1-B-B-B | EIAR | CIMMYT_ZIMBABWE |
| 62 | AML-62 | [B.T.Z.T.V.C -43-B -2-2 -1-1-#/F7215]-5-3-1-B-1-B-B-B | EIAR | CIMMYT_ZIMBABWE |
| 63 | AML-63 | [B.T.Z.T.V.C -43-B -2-2 -1-1-#/F7215]-6-1-2-B-2-B-B-B | EIAR | CIMMYT_ZIMBABWE |
| 64 | AML-64 | [[POOL9Ac7-SR(BC2)]FS60-2-1-3-2-1-#-#/142-1-E]-1-1-1-B-2-B-B-B | EIAR | CIMMYT_ZIMBABWE |
| 65 | AML-65 | [[POOL9Ac7-SR(BC2)]FS60-2-1-3-2-1-#-#/142-1-E]-1-1-2-B-1-B-B-B | EIAR | CIMMYT_ZIMBABWE |
| 66 | AML-66 | [B.T.Z.T.V.C -266-B-1-2 -2-1/F7215]-2-2-1-B-1-B-B-B | EIAR | CIMMYT_ZIMBABWE |
| 67 | AML-67 | [B.T.Z.T.V.C -266-B-1-2 -2-1/F7215]-3-1-1-B-1-B-B-B | EIAR | CIMMYT_ZIMBABWE |
| 68 | AML-68 | [B.T.Z.T.R.L .BA9 -3-1-1P-1-2P-1P-2-1-1 -1-B/142-1-e]-1-3-2-B-2-B-B-B | EIAR | CIMMYT_ZIMBABWE |
| 69 | AML-69 | [B.T.Z.T.R.L .BA9 -3-1-1P-1-2P-1P-2-1-1 -1-B/142-1-e]-2-2-1-B-1-B-B-B | EIAR | CIMMYT_ZIMBABWE |
| 70 | AML-70 | [B.T.Z.T.R.L .BA9 -3-1-1P-1-2P-1P-2-1-1 -1-B/142-1-e]-2-2-1-B-2-B-B-B | EIAR | CIMMYT_ZIMBABWE |
| 71 | AML-71 | [MORKA]-1-1-1-B-1-B-B-B | EIAR | CIMMYT_ZIMBABWE |
| 72 | AML-72 | [MORKA]-1-1-1-B-2-B-B-B | EIAR | CIMMYT_ZIMBABWE |
| 73 | AML-73 | [POOL9A C6 MHM-3-1-3-1-6-1P-2-2-2P-4-1-#/142-1-E]-1-3-1-B-1-B-B-B | EIAR | CIMMYT_ZIMBABWE |
| 74 | AML-74 | [POOL9A C6 MHM-3-1-3-1-6-1P-2-2-2P-4-1-#/142-1-E]-1-3-2-B-1-B-B-B | EIAR | CIMMYT_ZIMBABWE |
| 75 | AML-75 | [[KIT/SNSYN[N3/TUX]]c1F1-##(GLS=2)-1-1-2-2-#/F7215]-1-1-1-2-B-1-B-B-B | EIAR | CIMMYT_ZIMBABWE |
| 76 | AML-76 | [[KIT/SNSYN[N3/TUX]]c1F1-##(GLS=2)-1-1-2-2-#/F7215]-1-1-2-1-B-1-B-B-B | EIAR | CIMMYT_ZIMBABWE |
| 77 | AML-77 | [[KIT/SNSYN[N3/TUX]]c1F1-##(GLS=2)-1-1-2-2-#/F7215]-1-6-1-1-B-1-B-B-B | EIAR | CIMMYT_ZIMBABWE |
| 78 | AML-78 | [[KIT/SNSYN[N3/TUX]]c1F1-##(GLS=2)-1-1-2-2-#/F7215]-1-6-3-1-B-1-B-B-B | EIAR | CIMMYT_ZIMBABWE |
| 79 | AML-79 | [SINT T.SR.B.T.Z.T.8P-1P-1-5P-1P-1P-1-3-#/F7215]-1-2-3-1-B-2-B-B-B | EIAR | CIMMYT_ZIMBABWE |
| 80 | AML-80 | [SINT TSR.B.T.Z.T.19P-1P-1P-2P-1P-2-2-1/F7215]-1-1-3-2-B-2-B-B-B | EIAR | CIMMYT_ZIMBABWE |
| 81 | AML-81 | [B.T.Z.T.R.L -71 -B-3-3 -1-#-#/F7215]-1-2-1-1-B-1-B-B-B | EIAR | CIMMYT_ZIMBABWE |
| 82 | AML-82 | [{G9AC7HS 93-3/POOL 9A C6 MHM 3-1-3-1-1-2p-3p-4p-1-1-1-B-B)-1-1-1/F7215]-1-1-1-1-B-1-B-B-B | EIAR | CIMMYT_ZIMBABWE |
| 83 | AML-83 | [[ECU/SNSYN[SC/ETO]]c1F1-##(GLS=1.5)-3-1-2-2-#/142-1-E]-1-2-1-1-B-1-B-B-B | EIAR | CIMMYT_ZIMBABWE |
| 84 | AML-84 | [[KIT/SNSYN[N3/TUX]]c1F1-##(GLS=2)-35-2-1-2-#/142-1-E]-1-3-1-1-B-3-B-B-B | EIAR | CIMMYT_ZIMBABWE |
| 85 | AML-85 | [[POOL9Ac7-SR(BC2)]FS68-1-2-1-1-1-3-2/142-1-E]-1-3-1-1-B-2-B-B-B | EIAR | CIMMYT_ZIMBABWE |
| 86 | AML-86 | [B.T.Z.T.V.C -43-B -2-2 -1-1-#/142-1-E]-1-5-1-1-B-1-B-B-B | EIAR | CIMMYT_ZIMBABWE |
| 87 | AML-87 | [SRSYN95[ECU//SC/ETO]F1-##(GLS=3.5)-20-2-2-1-#-1/142-1-E]-1-2-1-1-B-2-B-B-B | EIAR | CIMMYT_ZIMBABWE |
| 88 | AML-88 | [SRSYN95[ECU//SC/ETO]F1-##(GLS=3.5)-20-2-2-1-#-1/142-1-E]-1-3-1-1-B-2-B-B-B | EIAR | CIMMYT_ZIMBABWE |
| 89 | AML-89 | [POOL9Ac7-SR(BC2)]FS59-4-1-2-1-1-1-#-#-#-# | EIAR | Parental line |
| 90 | AML-90 | [POOL9Ac7-SR(BC2)]FS67-1-2-3-1-#-#-#-#-# | EIAR | Parental line |
| 91 | AML-91 | [KIT/SNSYN[N3/TUX]]c1F1-##(GLS=1)-21-2-3-1-1-1-#-# | EIAR | Parental line |
| 92 | AML-92 | [KIT/SNSYN[N3/TUX]]c1F1-##(GLS=2)-32-2-2-1-1-#-# | EIAR | Parental line |
| 93 | AML-93 | [KIT/SNSYN[N3/TUX]]c1F1-##(GLS=2)-23-3-3-1-1-#-#-# | EIAR | Parental line |
| 94 | AML-94 | SRSYN95[ECU//SC/ETO]F1-##(GLS=3.5)-20-1-1-1-#-#-#-# | EIAR | Parental line |

AML**=**Ambo maize lines, EIAR=Ethiopian institute of agricultural research, CIMMYT= International Maize and Wheat Improvement Center
