# Supplementary material for: High-density single nucleotide polymorphism markers analysis reveals the genetic diversity and population structure in tropical highland maize (Zea mays L.) inbred lines
Source: PLoS One. 2026 Jun 22;21(6):e0351845. doi: 10.1371/journal.pone.0351845 (PMC13286140; doi:10.1371/journal.pone.0351845)
Supplement: S2 Table — (DOCX) [file pone.0351845.s003.docx]

Supporting Information Table S2

Table S2. Pairwise population Nm value among four germplasm source groups

|  | Group 1 | Group 2 | Group 3 | Group 4 |
| --- | --- | --- | --- | --- |
| Group 1 | 0.000 |  |  |  |
| Group 2 | 4.194 | 0 |  |  |
| Group 3 | 4.063 | 4.602 | 0 |  |
| Group 4 | 7.019 | 3.732 | 4.527 | 0 |

*N*m; number of migrants per generation.
