# Supplementary material for: High-density single nucleotide polymorphism markers analysis reveals the genetic diversity and population structure in tropical highland maize (Zea mays L.) inbred lines
Source: PLoS One. 2026 Jun 22;21(6):e0351845. doi: 10.1371/journal.pone.0351845 (PMC13286140; doi:10.1371/journal.pone.0351845)
Supplement: S3 Table — Proportion of ancestry (Q values) of each genotype assigned to the three inferred sub-populations. Genotypes with Q ≥ 0.60 were assigned to a specific cluster, while those with Q < 0.60 were considered admixed. These data correspond to the population structure illustrated in Figure 7. (DOCX) [file pone.0351845.s004.docx]

**Supporting Information S3**

**Table S3. Membership coefficients of 93 maize inbred lines inferred from population structure analysis (K = 3).** Proportion of ancestry (Q values) of each genotype assigned to the three inferred sub-populations. Genotypes with Q ≥ 0.60 were assigned to a specific cluster, while those with Q < 0.60 were considered admixed. These data correspond to the population structure illustrated in Figure 7.

| Number | Individuals | Subpopulation I | Subpopulation II | Subpopulation III |
| --- | --- | --- | --- | --- |
| 1 | AML1 | 0.675 | 0.140 | 0.185 |
| 2 | AML2 | 0.825 | 0.038 | 0.137 |
| 3 | AML3 | 0.928 | 0.001 | 0.071 |
| 4 | AML4 | 0.862 | 0.022 | 0.116 |
| 5 | AML5 | 0.812 | 0.001 | 0.187 |
| 6 | AML6 | 0.799 | 0.008 | 0.193 |
| 7 | AML7 | 0.867 | 0.023 | 0.110 |
| 8 | AML8 | 0.848 | 0.029 | 0.123 |
| 9 | AML9 | 0.798 | 0.012 | 0.190 |
| 10 | AML10 | 0.998 | 0.001 | 0.002 |
| 11 | AML11 | 0.799 | 0.032 | 0.169 |
| 12 | AML12 | 0.839 | 0.002 | 0.159 |
| 13 | AML13 | 0.852 | 0.001 | 0.147 |
| 14 | AML14 | 0.933 | 0.002 | 0.065 |
| 15 | AML15 | 0.993 | 0.003 | 0.004 |
| 16 | AML16 | 0.989 | 0.001 | 0.010 |
| 17 | AML17 | 0.994 | 0.001 | 0.005 |
| 18 | AML18 | 0.999 | 0 | 0.001 |
| 19 | AML19 | 0.995 | 0.001 | 0.004 |
| 20 | AML20 | 0.91 | 0.002 | 0.088 |
| 21 | AML21 | 0.925 | 0.008 | 0.067 |
| 22 | AML22 | 0.691 | 0.045 | 0.264 |
| 23 | AML23 | 0.574 | 0.126 | 0.301 |
| 24 | AML24 | 0.682 | 0.088 | 0.230 |
| 25 | AML25 | 0.712 | 0.105 | 0.182 |
| 26 | AML26 | 0.762 | 0.100 | 0.137 |
| 27 | AML27 | 0.688 | 0.038 | 0.274 |
| 28 | AML28 | 0.996 | 0.001 | 0.004 |
| 29 | AML29 | 0.001 | 0.001 | 0.998 |
| 30 | AML30 | 0.001 | 0.001 | 0.998 |
| 31 | AML31 | 0.001 | 0.001 | 0.999 |
| 32 | AML32 | 0.738 | 0.024 | 0.239 |
| 33 | AML33 | 0.717 | 0.024 | 0.259 |
| 34 | AML34 | 0.357 | 0.387 | 0.257 |
| 35 | AML35 | 0.286 | 0.323 | 0.391 |
| 36 | AML36 | 0.520 | 0.478 | 0.001 |
| 37 | AML37 | 0.130 | 0.870 | 0 |
| 38 | AML38 | 0.053 | 0.947 | 0 |
| 39 | AML39 | 0.032 | 0.967 | 0.001 |
| 40 | AML40 | 0.047 | 0.953 | 0 |
| 41 | AML41 | 0.787 | 0.039 | 0.174 |
| 42 | AML42 | 0.852 | 0.024 | 0.124 |
| 43 | AML43 | 0.274 | 0.447 | 0.28 |
| 44 | AML44 | 0.512 | 0.133 | 0.356 |
| 45 | AML45 | 0.512 | 0.135 | 0.353 |
| 46 | AML46 | 0.517 | 0.143 | 0.340 |
| 47 | AML47 | 0.991 | 0.004 | 0.005 |
| 48 | AML48 | 0.547 | 0.242 | 0.212 |
| 49 | AML49 | 0.447 | 0.284 | 0.269 |
| 50 | AML50 | 0.426 | 0.322 | 0.251 |
| 51 | AML51 | 0.313 | 0.049 | 0.639 |
| 52 | AML52 | 0.616 | 0.072 | 0.312 |
| 53 | AML53 | 0.994 | 0.001 | 0.006 |
| 54 | AML54 | 0.501 | 0.121 | 0.378 |
| 55 | AML55 | 0.918 | 0.001 | 0.081 |
| 56 | AML56 | 0.889 | 0.001 | 0.110 |
| 57 | AML57 | 0.996 | 0.002 | 0.001 |
| 59 | AML59 | 0.995 | 0.004 | 0.001 |
| 60 | AML60 | 0.733 | 0.084 | 0.183 |
| 61 | AML61 | 0.502 | 0.494 | 0.004 |
| 62 | AML62 | 0.281 | 0.544 | 0.176 |
| 63 | AML63 | 0.349 | 0.648 | 0.003 |
| 64 | AML64 | 0.449 | 0.550 | 0.001 |
| 65 | AML65 | 0.438 | 0.553 | 0.009 |
| 66 | AML66 | 0.244 | 0.007 | 0.749 |
| 67 | AML67 | 0.750 | 0.111 | 0.138 |
| 68 | AML68 | 0.186 | 0.022 | 0.792 |
| 69 | AML69 | 0.001 | 0.136 | 0.863 |
| 70 | AML70 | 0 | 0.134 | 0.866 |
| 71 | AML71 | 0.094 | 0.001 | 0.906 |
| 72 | AML72 | 0.056 | 0.001 | 0.943 |
| 73 | AML73 | 0.952 | 0.045 | 0.004 |
| 74 | AML74 | 0 | 0.375 | 0.625 |
| 75 | AML75 | 0.001 | 0.500 | 0.500 |
| 76 | AML76 | 0.001 | 0.269 | 0.730 |
| 77 | AML77 | 0.885 | 0.076 | 0.038 |
| 78 | AML78 | 0.952 | 0.044 | 0.004 |
| 79 | AML79 | 0.683 | 0.147 | 0.170 |
| 80 | AML80 | 0.914 | 0.024 | 0.062 |
| 81 | AML81 | 0.934 | 0.066 | 0 |
| 82 | AML82 | 0.394 | 0.447 | 0.159 |
| 83 | AML83 | 0.1 | 0.017 | 0.883 |
| 84 | AML84 | 0.461 | 0 | 0.539 |
| 85 | AML85 | 0.351 | 0.038 | 0.611 |
| 86 | AML86 | 0.027 | 0.426 | 0.547 |
| 87 | AML87 | 0 | 0.414 | 0.586 |
| 88 | AML88 | 0.300 | 0.029 | 0.671 |
| 89 | AML89 | 0.452 | 0.106 | 0.442 |
| 90 | AML90 | 0.735 | 0.068 | 0.197 |
| 91 | AML91 | 0.980 | 0.001 | 0.019 |
| 92 | AML92 | 0.987 | 0.001 | 0.012 |
| 93 | AML93 | 0.976 | 0.010 | 0.013 |
| 94 | AML94 | 0.720 | 0.023 | 0.257 |

AML= Ambo maize line
