# Supplementary material for: High-density single nucleotide polymorphism markers analysis reveals the genetic diversity and population structure in tropical highland maize (Zea mays L.) inbred lines
Source: PLoS One. 2026 Jun 22;21(6):e0351845. doi: 10.1371/journal.pone.0351845 (PMC13286140; doi:10.1371/journal.pone.0351845)
Supplement: Attachment — Submitted filename: ManuscriptPLOSone Review.doc [file pone.0351845.s005.doc]

***High-density Single Nucleotide Polymorphism markers analysis reveals the genetic diversity and population structure in tropical highland maize (Zea mays L.) inbred lines***

Worknesh Terefe^1*^, Demissew Abakemal^2^_,_ Tilahun	Mekonnen^3^, Tileye	Feyissa^3^

^1^Holeta Agricultural Research Center, Ethiopian Institute of Agricultural Research, Holeta, Ethiopia.

^2^Ambo Agricultural Research Center, Ethiopian Institute of Agricultural Research, Ambo, Ethiopia.

^3^Biotechnology Research Center, Addis Ababa University, Addis Ababa, Ethiopia.

*Correspondence: gworkneshterefe@gmail.com (WT)

**Abstract**

Genetic diversity is critical for crop improvement, germplasm conservation, and sustainable agriculture. It allows breeders to determine the relationships among germplasm, select parents, and develop resilient varieties. Eleven thousand two hundred and three (11,203) single nucleotide polymorphism (SNP) markers were used to assess the genetic diversity of 93 maize inbred lines adapted to the East African highlands. Gene diversity, polymorphic information content, and genetic distance ranged from 0.10-0.67, 0.10-0.59, and 0.03-0.52, with mean values of 0.46, 0.40, 0.44, respectively. linkage disequilibrium (LD) revealed 36,904 (7.6%) SNP pairs in significant LD (*r^2^* > 0.20, *p* < 0.01), with a mean *r*^2^ of 0.50. LD decay varied across chromosomes, from 0.26 Mb on Chromosome 4 to 5.41 Mb on Chromosome 7, with a genome-wide average of 3.33 Mb. Analysis of molecular variance showed 95% of the total variation occurred within populations, while 5% was among-population. Multivariate analyses including Neighbor joining, principal component analysis, and population structure consistently grouped the lines into three clusters, largely corresponding with pedigree information. The observed diversity indicates the presence of valuable alleles that can be harnessed in maize breeding to enhance productivity and resilience in highland environments. Furthermore, the identified SNPs represent a valuable genomic resource for marker trait association analysis to uncover the genomic regions underlying key agronomic traits and accelerate genetic improvement in challenging environments.

*Keywords*: Breeding; Clusters; DArTSeq; Genetic distance; Linkage disequilibrium

**Introduction**

Maize (*Zea mays* L.) is the third most important cereal crop globally after wheat and rice. It is widely cultivated for food, feed, and industrial purposes [1-3]. Global maize production increased from 313 million metric tons in 1971 to 1,162 million metric tons in 2020, reflecting its expanding role in global food systems. The United States, China, Brazil, the European Union, and Argentina are the leading producers [4].

In sub-Saharan Africa (SSA), maize is the most important cereal crop and serves as a primary energy source for over 300 million people, in addition to its uses in livestock feed and as industrial raw material [5-6]. Despite its significance, maize productivity in Ethiopia remains considerably below the global average yield of 5.88 t ha^-1^. This low productivity is partially attributed to the narrow genetic base created by prolonged selection within locally adapted germplasm [7-8]. Limited genetic diversity restricts breeding progress and reduces the potential for developing high-yielding, climate-resilient cultivars. Increasing population growth and rising food demand further highlight the need to explore and utilize existing genetic diversity for maize improvement.

Maize germplasm is broadly classified into temperate, subtropical, and tropical groups based on latitudinal and environmental adaptation [9]. Tropical maize generally exhibits higher allelic diversity than temperate germplasm [10-11], making it a valuable source of genetic resources for developing climate resilient cultivars [12]. Within the tropical group, maize is further categorized into lowland, midaltitude, and highland types. Highland maize is particularly notable for its superior performance in low-temperature conditions where other adaptation groups perform less [13]. Tropically adapted germplasm constitutes a major reservoir of genetic diversity [14-15]. Exploiting this diversity is essential for breeding programs targeting productivity, stress tolerance, and climate change adaptation.

Understanding genetic diversity and population structure is fundamental for effective crop improvement. Knowledge of genetic diversity enables breeders to identify divergent parental lines, maximize heterosis, and develop hybrids with enhanced resilience to environmental stress [16-17]. Analysis of population structure further enables the differentiation of breeding populations, the introgression of favorable alleles, and classification of inbred lines into heterotic groups, an essential step for hybrid maize development [18-20]. Collectively, these insights enable informed parent selection and support long-term genetic gains in maize improvement programs.

Molecular markers, particularly single nucleotide polymorphisms (SNP), are indispensable for assessing genetic diversity due to their abundance, genome-wide distribution, reproducibility, and suitability for high-throughput genotyping [21-22]. Advances in genotyping platforms, especially genotyping-by-sequencing (GBS), have dramatically enhanced the capacity of genome-wide SNP discovery and enabled detailed evaluation of genetic relationships, population structure, and linkage disequilibrium [23-24]. These high- resolution tools provide a robust foundation for characterizing germplasm diversity and accelerating the development of improved maize varieties.

In East Africa, recent breeding efforts have focused on developing highland-adapted maize inbred lines with improved productivity and resilience to cold stress, drought, and emerging diseases. Ethiopia possesses diverse maize germplasm, including unique highland-adapted types and yet few studies have employed high-density SNP markers to assess the genetic diversity of locally adapted inbred lines [25-26]. Earlier research relying on phenotypic traits or low-density markers provided limited resolution, leaving the genetic structure, subgroup classifications, and potential heterotic patterns largely unresolved.

Addressing this knowledge gap is crucial for strengthening national breeding programs. Detailed characterization genetic diversity enables the identification of complementary parents for hybrid development, minimizes redundancy in breeding materials, and improves selection efficiency. Insights into population structure also inform association mapping and genomic-assisted breeding strategies. In highland environments, where low temperatures and multiple stresses limit maize performance, well-characterized and genetically diverse inbred lines are essential to accelerate hybrid development. This study evaluated the genetic diversity and population structure of tropical highland-adapted maize inbred lines developed for East Africa using genome-wide SNP markers. Specifically, the study aimed to estimate key diversity parameters, examine population structure and sub-group formation and elucidate genetic relationships among lines to support parent selection and genomic-assisted breeding in highland maize improvement.

**Materials and methods**

**Plant materials**

A total of 93 maize inbred lines developed by the Ethiopian Institute of Agricultural Research (EIAR) for the highland agro-ecologies of Ethiopia were used (Table S1). These lines originated from both local and exotic germplasm through hybridization followed by successive selfing. Among them, 28 lines were derived from Ethiopian highland accessions, 26 were early-generation lines from CIMMYT Mexico, and 33 from CIMMYT Zimbabwe. Additionally, six lines were obtained from Ambo Agricultural Research Center (AARC). All lines were evaluated for adaptation and disease resistance, and those with superior performance under optimal conditions were selected for this study.

**DNA extraction and SNP genotyping**

Genomic DNA was extracted from fresh leaf tissues of three-week-old seedlings grown under greenhouse conditions at Melkasa Agricultural Research Center. Samples were collected in 96 deep-well plates, freeze-dried, and extracted using the Nucleomag Plant Genomic DNA Extraction Kit (Macherey-Nagel GmbH & Co. KG, Duren, Germany), following the DArT protocol [27]. DNA quality and concentration were verified using a NanoDrop^TM^ 2000 Spectrophotometer (Thermo Scientific^TM^, USA) and 0.8% agarose gel electrophoresis. Genotyping was performed using the genotyping-by-sequencing (GBS) method as described by Elshire, Glaubitz [28]. Genomic DNA was digested with ApeKI barcoded adapters were ligated, and fragments were PCR amplified. The libraries were sequenced as 77 bp single-end reads on an Illumina HiSeq 2500 system (Illumina, San Diego, CA, USA).

**SNP data filtering and analysis**

Raw SNP data were filtered to exclude markers with polymorphic information content (PIC) < 0.1, heterozygosity < 0.02 [29], minor allele frequency (MAF) < 0.05, and missing data > 30%. Genetic diversity parameters including MAF, heterozygosity (He), gene diversity (GD), and PIC were calculated using PowerMarker v3.2.5 [30]. Genetic distances were estimated using Nei’s method Nei and Takezaki [31], and a neighbor-joining (NJ) phylogenetic tree was constructed and visualized in MEGA v11 [32]. Pairwise linkage disequilibrium (LD) was estimated using the squared correlation coefficient (r^2^) in TASSEL v5.2.8 [33] with an LD window size of 50 bp. Only SNP pairs with significant LD (r^2^ ≥ 0.2, p < 0.1) were retained. LD decay was visualized using a locally weighted scatterplot smoothing (LOESS) curve in R, [34] and the decay rate was defined as the physical distance at which r^2^ declined to half its maximum average [35].

Analysis of molecular variance (AMOVA) was conducted in GenAlEx v6.5 [36] following Excoffier, Smouse [37] and [38]. Principal component analysis (PCA) was performed using the *prcomp()* function in R v4.4.1 [34], and visualized with *ggplot2* [39]. Population structure was inferred using STRUCTURE v2.3.4 [40] with an admixture model and correlated allele frequencies, using 10,000 burn-in and 50,000 MCMC iterations across K = 1-10. The optimum value was determined using the Evano method [41] via STRUCTURE HARVESTER [42]. Inbred lines with membership probabilities > 60% were assigned to a specific cluster, while those with < 60% were classified as admixed.

**Results**

**DArTseq marker characteristics and distribution**

Assessing genetic diversity is a fundamental step in plant breeding, as it provides a foundation for developing high-yielding, stable, and stress-tolerant genotypes that ensure food and nutritional security. Genotyping of the 93 maize inbred lines using the DArTseq platform initially generated 31,316 SNP markers. After quality control (QC) filtering, 11,203 high-quality markers retained, 9,770 markers successfully aligned to maize reference genome, and 1,433 mapped to unknown positions. The SNPs were distributed across all ten chromosomes, with chromosome 1 containing the highest number (1,491) and chromosome 10 the fewest (637), average 977 SNPs per chromosome (Fig 1).

Fig 1. SNP distribution across ten chromosomes of maize inbred lines

**Levels of SNP Polymorphism**

Genome-wide diversity indices revealed substantial variation among loci. Polymorphic information content (PIC) values ranged from 0.10 to 0.59, with mean value of 0.40, showing that the SNPs used were highly informative and suitable for marker-trait association analyses. Among the SNPs, 14% exhibited low PIC (< 0.3), 50% moderate (0.3-0.4), and 36% high (> 0.5) polymorphism (Fig 2B). Allele frequencies ranged from 0.05 to 0.95 (Table 1; Fig 2A). Locus heterozygosity (He), gene diversity (GD), PIC, and minor allele frequency (MAF) showed slight variation across chromosomes (Fig 3). Heterozygosity ranged from 0.04 to 0.05, with chromosomes 9 and 10 exhibiting the lowest values. Pairwise genetic distances among the inbred lines ranged from 0.03 to 0.52, averaging 0.44 (Table 1). The greatest genetic distance was observed between AML70 and AML2 (additional file1), derived from different populations, while the smallest occurred between closely related sister lines AML31 and AML30.

Fig 2. Frequency distribution of (A) minor allele frequency (MAF) and (B) polymorphic information content (PIC) of 11,203 DArTSeq SNP markers

Fig 3. Distribution of summary statistics (MAF, He and GD) of the 11,203 SNPs on the 10 chromosomes of all inbred lines

Table 1 Level of polymorphism of 11,203 SNPs markers in 93 maize inbred lines

| Statistics | MAF | He | GD | PIC | GDist |
| --- | --- | --- | --- | --- | --- |
| Minimum | 0.05 | 0.04 | 0.10 | 0.10 | 0.03 |
| Maximum | 0.95 | 0.05 | 0.67 | 0.59 | 0.52 |
| Mean | 0.34 | 0.04 | 0.46 | 0.40 | 0.44 |

MAF=Minor allele frequency, He= Heterozygosity, GD= Gene diversity, PIC= Polymorphic information content, GDist= Genetic distance

**Allelic diversity within-population**

Genetic diversity indices revealed pronounced differences within populations but limited variation among them (Table 2). The mean observed number of alleles (Na = 1.502), effective number of alleles (Ne = 1.205), expected heterozygosity (He = 0.153), and Shannon’s information index (I = 0.318) indicated moderate diversity. Among the four populations, Population 1 showed the highest diversity (Na = 1.70, Ne = 1.313, He = 0.203, I = 0.318, and 70.33% polymorphic loci), suggesting a broad genetic base and balanced allele distribution. Conversely, Population 4 displayed reduced diversity (Na = 1.10, He = 0.097, and 22.66% polymorphic loci).

Table 2 Summary of genetic diversity statistics across-loci for each population

| Population | Na | Ne | Ho | He | I | (%P) |
| --- | --- | --- | --- | --- | --- | --- |
| Pop1 | 1.70 | 1.31 | 0.00 | 0.20 | 0.32 | 70.33 |
| Pop2 | 1.62 | 1.26 | 0.00 | 0.18 | 0.28 | 62.96 |
| Pop3 | 1.56 | 1.20 | 0.00 | 0.14 | 0.23 | 59.16 |
| Pop4 | 1.10 | 1.05 | 0.00 | 0.10 | 0.14 | 22.66 |
| Overall | 1.50 | 1.21 | 0.00 | 0.15 | 0.32 | 53.73 |

Na =Number of Alleles, Ne= effective number of alleles, Ho=Observed Heterozygosity, He= Expected Heterozygosity, I= Shannon’s Index, (%P) = Polymorphic loci

**Linkage disequilibrium (LD) analysis**

Genome-wide LD was assessed using pairwise r^2^ values across the ten chromosomes. A total of 558,876 marker pairs were analyzed, yielding a mean r^2^ = 0.50, with 36,904 pairs (7.6%) showing significant LD at p ≤ 0.01 (Table 3). Chromosome 1 containing the highest number of marker pairs (73.275), followed by chromosome 2 (61,899), whereas chromosomes 10 had the fewest (31,850). The strongest chromosome-wise LD was recorded on chromosome 3 (r^2^ = 0.78). Genome-wide, LD decayed to r^2^ = 0.2 within 3.33 Mb (Fig 4), with chromosome-specific decay ranged from 0.26 Mb (Chromosome 4) to 5.41 Mb (Chromosome 7). Higher LD on chromosome 7 may reflect reduced recombination or selection sweeps, while faster decay on chromosome 4 suggests greater haplotype diversity and recombination activity. As expected, closely spaced SNPs exhibited higher LD, which declined with increasing physical distance, reflecting recombination effects across the genome.

Fig 4. Scatter plot of genome-wide LD decay against total physical distance (bp). The horizontal blue line represents half decay r^2^ value of genome (r^2^ = 0.2). Red curve line is the smoothening spline regression model fitted to LD decay

Table 3 Summary of linkage disequilibrium analysis among marker pairs and the number of significant pairs per chromosome.

| Chromosome | TMNP | r^2^ | Distance (Mp) | Significant marker pairs (*P* < 0.01) |
| --- | --- | --- | --- | --- |
| 1 | 73275 | 0.01 | 4.81 | 5071(6.9%) |
| 2 | 61899 | 0.5 | 1.71 | 5668 (9.19%) |
| 3 | 53100 | 0.78 | 3.89 | 3628 (6.8%) |
| 4 | 49600 | 0.07 | 0.26 | 2943 (5.9%) |
| 5 | 56600 | 0.02 | 2.64 | 4658(8.2%) |
| 6 | 39150 | 0.5 | 3.02 | 3 (0.0%) |
| 7 | 40150 | 0.01 | 5.41 | 2527(6.3%) |
| 8 | 43350 | 0.51 | 4.82 | 3450(8.0%) |
| 9 | 38250 | 0.27 | 3.09 | 3486(9.1%) |
| 10 | 31850 | 0.01 | 3.61 | 2428(7.6%) |
| Total | 558876 | 0.5 | 3.33 | 36904(7.6%) |

*TNMP, total number of marker pairs; Mp, mega-base pairs.*

**Analysis of molecular variance (AMOVA)**

AMOVA revealed that 95% of the total genetic variance was partitioned within populations and only 5% among them (Table 4). This pattern corresponds with the low overall F_ST_ value (0.05), indicating limited genetic differentiation among populations and substantial within-population variation. The overall genetic differentiation was low (PhiPT = 0.01, *p* = 0.0001), consistent with a high level of gene flow (Nm = 4.35; Table S2). These findings suggest extensive allele exchange and shared ancestry among the inbred lines.

Table 4 Analysis of molecular variance (AMOVA) in four maize populations based on high density DArTSeq SNPs

| Source | DF | SS | MS | Estimate of Variance components | % of Variation |
| --- | --- | --- | --- | --- | --- |
| Among Pops | 3 | 56187.16 | 18729.05 | 478.44 | 5 |
| Within Pops | 89 | 741400.813 | 8330.35 | 8330.35 | 95 |
| Total | 92 | 797587.968 |  | 8808.78 | 100 |

Genetic differentiation among populations and within groups (inbred lines) PhiPT= 0.01; p=0.0001; P-value is based on 9999 permutations. DF= degree of freedom; SS= sum of squares; MS= mean of squares.

**Population differentiation**

Pairwise F_ST_ estimates among the four sub-populations were uniformly low, ranging from 0.001 to 0.009 (Table 5). Sub-populations 1, 2, and 3 exhibited the least differentiation from one another. Comparisons involving sub-population 4 showed the highest differentiation. Although the F_ST_ values remain within the range denoting minimal divergence, Nei’s population genetic distance values supported these patterns, spanning from 16,440 to 17,602. The smallest distance was observed between sub-populations 1 and 2, whereas the greatest distance occurred between sub-populations 1 and 4. The overall F_ST_ for the panel was 0.05. The corresponding gene flow estimate (Nm = 4.35) indicated that, on average, more than four migrants per generation contribute to genetic exchange among the sub-populations, thereby limiting population differentiation.

Table 5 Pairwise population F_ST_ values calculated for the four sub-populations above diagonal and pairwise population genetic distance below diagonal

| Sub-populations | 1 | 2 | 3 | 4 |
| --- | --- | --- | --- | --- |
| 1 | 0 | 0.001 | 0.001 | 0.008 |
| 2 | 16440 | 0 | 0.001 | 0.009 |
| 3 | 16558 | 17328 | 0 | 0.007 |
| 4 | 17602 | 16769 | 17446 | 0 |
| Overall F_ST_ value | 0.05 | | | |
| Overall Nm value | 4.35 | | | |

**Clustering and population structure**

Neighbor-joining (NJ), population structure, and principal component analysis (PCA) consistently revealed three genetic subgroups, reflecting distinct gene pools or evolutionary backgrounds. The NJ dendrogram grouped the 93 maize inbred lines into three main clusters (C1-C3) (Fig 5). Cluster 1 contained the largest germplasm (54 lines, 58%), predominantly of exotic origin, though it also included three Ethiopian highland lines (AML20, AML27, AML94). Cluster 3 comprised 38 lines (40.4%), mainly derived from the Kitale and F7215 groups. The clustering pattern generally corresponded with pedigree relationships, with sister lines grouped together, reflecting shared lineage. For instance, lines from the AMB16N37-LD group were derived from testers as F7215 (Kitale origin) and 142-1-e (Ecuador origin), indicating the diverse ancestry of the germplasm.

Fig 5. Phylogenetic tree generated from pairwise genetic distance using the neighbor joining (NJ) method

Principal component analysis (PCA) confirmed three genetic clusters (Fig 6). The first two principal components (PC1 and PC2) explained 8.81% of the total variation (4.7% and 4.11%, respectively). Lines from population 1 were exclusively grouped in cluster 1, whereas those from other populations were distributed across the remaining clusters, suggesting derivation from diverse parental crosses.

Fig 6. Principal component analysis based on 11,203 SNP markers revealed the four populations into three major groups. Samples coded with the same color belong to the same population

Population structure analysis using STRUCTURE software also supported the presence of three subpopulations, with a distinct peak in ΔK at K = 3 (Fig 7A, B). Based on a membership threshold > 0.60, 51 lines (54.8%) were assigned to subpopulation 1, five (5.4%) to subpopulation 2, and 15 (16.1%) to subpopulation 3 (Table S3). The remaining 22 lines (23.7%) were classified as admixed. While NJ, PCA, and STRUCTURE analyses produced largely concordant clustering, minor discrepancies in line assignment were noted, likely due to genomic admixture among populations.

Fig 7. Population structure of 93 maize inbred lines using 11,203 SNPs, (A) best delta K estimated by Evanno method for K = 3, (B) estimated structure of 93 tropical maize inbred lines. The different colors (red, green, and blue) represent subpopulations 1, 2, 3

**Discussion**

The assessment of genetic diversity and population structure provides essential insights into the evolutionary relationships, breeding potential, and adaptability of maize germplasm. In the present study, the observed patterns of genetic variation reveal substantial diversity among the lines, indicating the presence of potentially valuable alleles that can be exploited for future breeding programs. In maize, such diversity is crucial for exploring heterosis through hybrid development, which depends on crossing germplasm from divergent clusters [43-45]. Identifying breeding materials carrying desirable alleles and mapping these alleles to target traits are therefore, vital for efficient selection and hybrid formation.

Relatively higher PIC values observed in the present study indicate a greater level of allelic diversity and informativeness of the markers used. This suggest that the maize panel harbors more genetic variation than previously reported populations by Ayesiga, Rubaihayo [23], Sserumaga, Makumbi [46], Stanley, Menkir [47]. This suggests a broad genetic base that can be exploited to select diverse parents, enhance marker-assisted selection efficiency, and accelerate genetic improvement in maize breeding. The high gene diversity and wide genetic distances among lines indicate a broad genetic base, essential for achieving high heterosis in hybrid combinations. Only two pairs of lines showed genetic distances below 0.05, suggesting minimal redundancy and wide variation, the present finding is comparable to the findings of Ertiro, Semagn [48], Semagn, Magorokosho [45]. Comparable diversity levels were observed in Ethiopian highland maize accessions [26], supporting the uniqueness of the lines evaluated in this study.

Genetic diversity indices revealed pronounced differences within populations but limited variation among them (Table 3). The mean observed number of alleles (Na = 1.502), effective number of alleles (Ne = 1.205), expected heterozygosity (He = 0.153), and Shannon’s information index (I = 0.318) indicated moderate diversity which is consistent with earlier studies in tropical maize [49]. Among the four populations, Population 1 showed the highest diversity, suggesting a broad genetic base and balanced allele distribution. Such diversity makes it a valuable source of alleles for breeding and hybrid development [50]. Conversely, Population 4 displayed reduced diversity, possibly due to genetic bottlenecks, selection pressure, or restricted gene flow [51]. Overall, Population 1 serves as a valuable source of genetic variation, while Population 4 may require enrichment through introgression of diverse germplasm. These findings emphasize the importance of conserving genetically diverse populations, particularly Population 1 to ensure sustained genetic gain and adaptability in maize improvement.

The rapid decay of LD is crucial for association studies as it allows for the identification of genes contributing to phenotypic variation with high precision. Previous studies have reported wide ranges in LD (r^2^ = 0.14-0.75) depending on number of populations, structure, and marker distribution [52-53]. The present findings are consistent with those of Amadu, Beyene [54], and Kumar, Rakshit [52] confirming the dynamic recombination landscape typical of maize. Understanding LD decay provides valuable insight for determining marker density requirements in genome-wide association studies (GWAS) and designing efficient marker-assisted selection (MAS) strategies.

The predominance of within-population variation indicates that the maize inbred lines share considerable genetic similarity, likely resulting from recombination, mutation, and historical gene exchange. Similar patterns were observed by Ayesiga, Rubaihayo [23] and Sserumaga, Makumbi [55], who reported 97-98% of variation within maize inbred populations. The high gene flow observed (Nm = 4.35) further supports limited population differentiation and gene exchange. Conversely, Nelimor, Badu-Apraku [51] reported lower Nm values in maize landraces, suggesting restricted gene flow compared with inbred lines. Nonetheless, the genetic distance observed between sub-populations, particularly between populations 1and 4, may provide opportunities to exploit heterosis in breeding programs.

The F_ST_ values (0.015-0.716) were consisted with previous reports [23] [55], indicating strong differentiation among lines. These high F_ST_ values suggest that most inbred lines are genetically distinct and largely fixed, suitable for heterotic grouping. The low heterozygosity typical of advanced maize inbreds [44].

The congruence among NJ, PCA, and STRUCTURE analyses indicates a well-defined yet interconnected genetic structure among the maize inbred lines, reflecting their diverse ancestral origins and breeding histories. The presence of three distinct subgroups suggests contributions from multiple gene pools, likely arising from the integration of exotic and Ethiopian germplasm during inbred line development. The clustering of Ethiopian lines alongside exotic materials highlights the genetic intermixing that has occurred through historical introgression and selection, processes that enhance adaptability to diverse agro-ecological conditions as indicated by Blanco-Pastor [56],He, Pasam [57].

The observed admixture, supported by overlapping clusters and low differentiation indices (F_ST_ and AMOVA), reflects extensive gene flow and shared ancestry expected in breeding programs utilizing CIMMYT derived materials. This genetic blending is advantageous as it broadens the allelic base, increases recombination potential, and reduces the risk of inbreeding depression. The 23.7% admixture rate aligns with previous reports [58-59] and underscores the dynamic nature of maize gene exchange due to open pollination and the recurrent use of common parents.

From a breeding perspective, the differentiation between clusters, particularly between populations 1 and 4, provides opportunities for heterotic hybrid formation. The diverse ancestry represented in these subpopulations can be harnesses to combine complementary alleles for yield, stress tolerance, and disease resistance aligns with the earlier reports [60-61]. Overall, the structured yet interconnected diversity observed in this study confirms that the evaluated maize inbred lines constitute a rich genetic reservoir for association mapping and marker-assisted selection, contributing to the development of resilient, high-yielding cultivars adapted to varied environments.

**Conclusion**

This study demonstrated substantial genetic diversity among 93 tropical maize inbred lines based on high-density SNP markers, with an average PIC value of 0.40 and wide pairwise genetic distances. The lines were clearly structured into three distinct genetic clusters, reflecting a robust population structure. These results underscore the richness of the maize gene pool developed for tropical highland conditions. The identified inbred lines constitute favorable genetic resources harboring favorable alleles for breeding programs, providing a solid foundation for genome-wide association studies, trait improvement, and efficient conservation and utilization of maize genetic diversity.

**Acknowledgments**

The authors are grateful for the Ethiopian Institute of Agricultural Research (EIAR), particularly to Ambo Agricultural Research Center for providing the maize inbred lines used in this study. We also sincerely thankful for Bill & Melinda Gates Foundation for financial support in genotyping of the inbred lines under the Modernizing Ethiopian Research on Crop Improvement (MERCI) Project.

**References**

**Supporting information caption**

Additional file: Nei’s genetic distance matrix among 93 maize inbred lines

**Supplementary tables**

Table S2. List of maize inbred lines and their pedigree information used for genetic diversity

Table S3. Pairwise population Nm value among four sub-populations

Table S4. Proportion of membership of 93 maize inbred lines revealed by population structure analysis
