# Supplementary material for: High-density single nucleotide polymorphism markers analysis reveals the genetic diversity and population structure in tropical highland maize (Zea mays L.) inbred lines
Source: PLoS One. 2026 Jun 22;21(6):e0351845. doi: 10.1371/journal.pone.0351845 (PMC13286140; doi:10.1371/journal.pone.0351845)
Supplement: Attachment — Submitted filename: PONE REVIEW.docx [file pone.0351845.s006.docx]

The manuscript tilted “High-density Single Nucleotide Polymorphism markers analysis reveals the genetic diversity and population structure in tropical highland maize (Zea mays L.) Inbred lines” is well written scientifically and analytically. Also, it contributes to the understanding of tropical highland maize germplasm for improvement. The manuscript could benefit more by using some of the comments below.

**Abstract**

Line 52: Clarify whether the mean r^2^ of 0.50 is that of the significant pairs or genome-wide mean across all pairs.

Line59 – 62: You said “Furthermore, the identified SNP……” what SNPs were identified?

**Introduction**

Line 114: Add the preposition “of” “Detailed characterization **of**……”

Line 68—123: the introduction needs the inclusion of study hypothesis.

**Materials and Methods**

**Planting materials**

Line 131—133: do you mean to say the 93 lines were the selected following performance evaluation from the pool of inbreds? if so, please rephrase the sentence. The current form gives the impression that 93 lines were evaluated and the best performers were selected.

**Statistical Analysis**

Line 146—147: a. Use filtered for PIC and MAF. This is redundancy as both PIC and MAF exhibit near perfect collinearity. Just one of these is okay. Also I assume you mean to say “Raw” and not “Row”

b. The rationale for filtering SNPs with heterozygosity < 0.02 is unclear for inbred germplasm. For inbred maize lines, low heterozygosity is expected. Why remove low-heterozygosity SNPs? Does this bias diversity estimates? Normally, SNPs with high heterozygosity (genotyping errors) are removed not low heterozygosity.

c. In addition, you used >30% for missing data threshold, don’t you think this may introduce noise? Why don’t you tighten this to 10 or 20%?

Line 151—152: the authors said “Pairwise linkage disequilibrium (LD) was estimated using the squared correlation coefficient (r2 152 ) in TASSEL v5.2.8 [33] with an LD window size of 50 bp”. For maize SNP datasets, LD is typically evaluated over kilobase to megabase scales. Why restrict to 50 bp? Does this capture meaningful LD structure? Is this a typo for 50 kb or 500 kb? In my opinion, the LD window size of 50 bp is unusually small for maize and may bias LD estimation.

Line 153: the authors wrote p < 0.1 but earlier in the abstract, you reported p < 0.01. which is correct? 0.1 is very lenient for LD and will increases the chances for false positives.

Line 161 – 163: I will suggest you increase membership probability levels to a minimum of 0.70 or at 0.80. You are dealing with inbred lines and not clonal crops. Don’t you think at 0.6 may inflate pure group assignments? Burn-in of 10K is good but more reliable if you increased it to 50k-100K. Same for MCMC, good at 50K but more reliable at 100K-500K.

**Results**

Line 170: the authors did not make it clear that ~13% of the 11,203 markers were mapped to unknown position of the genome prior to the results section. Were these markers also involved in downstream analyses like LD analysis?

Line 191: font size issue need fixing for the table title.

Line 194—201: there seems to be a confusion that requires clarification. Which population are the authors making inference to here? Does this mean the four germplasm sources? The abstract clearly declared three clusters which is contradicting to four populations.

Line 205—216: Across all pairwise SNPs, a mean of 0.50 is unrealistically high in maize. Was this calculated only among significant pairs? Was LD computed after your heavy filtering? Is there population structure inflation? Or Is this a reporting mistake? The expected ranges in maize typical for genome-wide mean r² in diverse panels is between 0.02–0.10, for elite panels is 0.05–0.25. Though for biparental this may be higher but not up to 0.50.

Line 226: The Fst of 0.05 is not entirely low as you have interpreted. It is at the boundary of low to moderate differentiation. The conventional scale in population genetics <0.05 → little genetic differentiation; 0.05–0.15 → moderate differentiation; 0.15–0.25 → great differentiation; and > 0.25 → very great differentiation.

Line 237: I don’t think you need another caption for “population differentiation”. It is single analysis pipeline in GenAlex. You get your overall Fst/PhiPT values which is the same as your between group variation but expressed on a 0 to 1 scale. For example, your between population variation was 5%, if you convert that to 0 to 1 scale by dividing by 100, you get 0.05 as overall Fst/PhiPT value. Now when you break down this overall values into the population pairs you get a matrix table of the pairwise Fst (lower diagonal) and their respective probability level (upper diagonal) which you reported in Table 5. So you can just continue your interpretation without a new caption.
