# Supplementary material for: High-density single nucleotide polymorphism markers analysis reveals the genetic diversity and population structure in tropical highland maize (Zea mays L.) inbred lines
Source: PLoS One. 2026 Jun 22;21(6):e0351845. doi: 10.1371/journal.pone.0351845 (PMC13286140; doi:10.1371/journal.pone.0351845)
Supplement: Attachment — Submitted filename: Response to Reviewers.docx [file pone.0351845.s008.docx]

**Response to reviewers**

We sincerely thank the Editor and Reviewers for their careful evaluation and constructive comments. Their suggestions substantially improved the clarity, methodological rigor, and overall quality of the manuscript. All revisions in the manuscript are highlighted in bold text.

We have carefully revised the manuscript in response to all comments. Major revisions include:

- Reanalysis and correction of linkage disequilibrium (LD) results and interpretation,
- Improvement of methodological descriptions and SNP filtering criteria,
- Revision of population structure and genetic diversity interpretations,
- Correction of inconsistencies in *F*_ST_ reporting,
- Improvement of figure quality, table formatting, and supplementary information,
- Clarification of terminology distinguishing predefined germplasm source groups from genetically inferred clusters,
- Revision of language, grammar, and scientific clarity throughout the manuscript, and
- Deposition of all datasets in the Figshare Repository in accordance with PLOS ONE data availability requirements.

In addition, all statistical analyses, particularly LD estimation and population differentiation analyses, were carefully re-evaluated and verified to ensure consistency and accuracy throughout the manuscript.

**Reviewer #1**

**Comment 1**. Lines 127-129 can be elaborated for development of the inbred lines

**Response:** Additional details were added describing the origin and development of the inbred lines.

**Comment 2.** Clarify adaptation and disease resistance strategies.

**Response:** The manuscript now clarifies that adaptation and disease resistance strategies during line development.

**Reviewer #2**

**Comment 1.** Provide high-resolution figures.

**Response:** All figures were replaced with high-resolution versions (≥ 300 dpi), and figure legends were revised for improved clearly and readability.

**Comment 2.** The abstract should be more informative.

**Response:** The abstract was revised to improve clarity and informativeness by correcting LD statistics, refining diversity descriptions, and revising the concluding statements.

**Comment 3.** If possible, the Gst and Rst value may be added.

**Response:** Because the present study used biallelic SNP markers, AMOVA and FST based approaches were considered more appropriate and are widely applied in SNP based population genetic studies. Therefore, the manuscript retains the AMOVA-based estimates of genetic differentiation.

**Comment 4.** Authors should see if the SNPs can be associated with specific traits.

**Response**: GWAS framework was beyond the scope of the present study. Nevertheless, the discussion now emphasizes that the SNP dataset provides a valuable resource for future marker-trait association studies and genomic-assisted breeding.

**Reviewer #3**

**Comment 1.** The LD decay analysis mentions a window size of 50 bp-this seems unusual

**Response:** The previously reported “50 bp” value resulted from misinterpretation of the TASSEL parameter. In TASSEL, the LD window refers to the number of adjacent markers rather than physical distance. LD was recalculated using physically mapped SNPs, LOESS smoothing, and 10 kb distance bins. The revised analysis estimated genome-wide LD decay at approximately 93.82 kb (*r*² = 0.2), consistent with previous maize studies. All related sections, figures, and tables were updated accordingly

**Comment 2.** The STRUCTURE analysis used relatively low burn-in and MCMC iterations.

**Response:** The STRUCTURE analysis used 10,000 burn-in iterations and 50,000 MCMC repetitions. Multiple independent runs produced stable clustering patterns consistently supported by STRUCTURE, PCA, and neighbor-joining analyses. This justification has been clarified in the manuscript.

**Comment 3.** The PCA explains only ~8.8% of the total variation.

**Response:** The Discussion section has been revised to clarify that PCA alone may have limited power to resolve complex population structure, and therefore complementary analyses including STRUCTURE and neighbor-joining clustering were used to provide a more comprehensive assessment.

**Comment 4.** Table 3 shows chromosome 6 with 0 significant LD pairs.

**Response:** This resulted from a reporting error. After correction, chromosome 6 contained 3,027 significant LD pairs (r² ≥ 0.2). Table 3 and related text were updated.

**Comment 5.** Discuss low diversity in population 4 and the implications of LD decay for GWAS.

**Response:** The Discussion was expanded to explain that the lower diversity observed in germplasm source group 4 is likely associated with selection history and genetic drift, which may have reduced allelic variation through recurrent breeding and shared ancestry. The implications of the observed LD decay (~93.82 kb) for GWAS resolution were also discussed.

**Comment 6.** Authors must deposit the SNP data in a public repository

**Response:** The Data Availability Statement does not comply with PLOS ONE policy.

**Response:** All genotype datasets and supporting files were deposited in the Dryad Digital Repository and are publicly accessible at: <https://doi.org/10.6084/m9.figshare.32396424>

**Minor Comments**

**Response:** All figures have been revised to ensure clear axis labeling and inclusion of appropriate units. Table headings and supplementary materials were corrected and standardized throughout the manuscript. Formatting inconsistencies, spacing, statistical notation, and terminology were also revised for consistency.

**Comment.** In Abstract: The sentence missing a verb should be clarified.

**Response:** The sentence has been revised to include the appropriate verb for clarity.

**Comment.** Consider adding "GWAS" or "Genomic selection" if relevant to future applications.

**Response:** The keywords section has been revised to include GWAS.

**Comment.**  Additional File S1, and the Supporting Information section must be corrected too.

**Response:** The formatting has been corrected to “Additional File S1,” and the Supporting Information section has been revised accordingly.

**Comment.** "Cluster 1 contained the largest germplasm (54 lines, 58%)" – rephrase it

**Response:** The sentence has been revised and improved.

**Comment.** The sentence is a general statement; consider moving to the Introduction

**Response:** The sentence has been shortened to improve conciseness while maintaining its relevance in the Discussion section.

**Comment.** Check for inconsistent spacing and consistent italic formatting for statistical symbols.

**Response:** We have corrected the spacing and standardized the formatting of statistical symbols (p, *F*_ST,_ *and r*²) throughout the manuscript.

**Comment.**  Table 5 formatting confusion

**Response:** Table 5 was reformatted to improve clarity and consistency of pairwise FST presentation.

**Reviewer #4**

**Comment.** The manuscript contains numerous grammatical, typographical, and syntactic errors.

**Response:** The manuscript was thoroughly revised for grammar, syntax, and readability. Repetitive and ambiguous statements were corrected, and the manuscript underwent comprehensive English language editing.

**Comment.** SNP filtering criteria lack justification.

**Response.** The manuscript now provides justification and supporting references for SNP filtering criteria. Markers with low MAF, excessive missing data, and high heterozygosity (> 0.02) were removed to improve marker quality and analytical robustness.

**Comment.** The LD window size of 50 bp is unrealistic.

**Response:** This issue was addressed earlier (Reviewer #3, Comment 1). Briefly, the “50 bp” value resulted from misinterpretation of the TASSEL parameter; the analysis used a window of 50 adjacent markers rather than physical distance.

**Comment.** STRUCTURE iterations appear low.

**Response:** This issue was addressed earlier (Reviewer #3, Comment 2). The selected burn-in and MCMC values produced stable and reproducible clustering patterns across independent runs. This clarification was added to the manuscript.

**Comment.** The reported LD decay (~3.33 Mb) is inconsistent with maize studies.

**Response:** The LD analysis was re-evaluated using only physically mapped SNPs. The corrected genome-wide LD decay estimate was approximately 93.82 kb (*r*^2^ = 0.2), consistent with previous maize studies. All related analyses, figure, and interpretations were revised accordingly.

**Comment.** The biological and breeding significance of clusters is not adequately discussed.

**Response:** The Discussion was expanded to relate the identified clusters to pedigree background, germplasm origin, adaptation, and breeding history, as well as their implications for maize improvement and heterotic grouping.

**Comment.** The manuscript reports contradictory *F*_ST_ values in different sections.

**Response:** We carefully rechecked the analysis. Pairwise *F*_ST_ values ranged from 0.001 to 0.009, indicating low to moderate differentiation.

**Comment.** The Data Availability Statement does not comply with PLOS ONE policy.

**Response:** This was addressed earlier (Reviewer #3, Comment 6).

**Comment.** Improve figure quality legends.

**Response:** All figures were improved and was addressed earlier (Reviewer #2, Comment 1)

**Comment.** Some tables contain formatting inconsistencies.

**Response:** All tables were reformatted for consistency in style, spacing, alignment, and notation.

**Comment.** Citations should be updated and formatted consistently.

**Response:** All citations and references were reviewed and standardized according to journal guidelines using EndNote.

**Reviewer #5**

**Comment 1.** Clarify the mean *r*^2^ of 0.50

**Response:** The revised manuscript now reports a genome-wide mean *r*^2^ value of 0.067 across all pairwise SNP comparisons. This was addressed earlier (Reviewer #3, Comment 1).

**Comment 2.** Lines 59-62: furthermore, the identified SNP…what SNPs were identified?

**Response:** The statement was revised for clarify. The text now refers to the SNP markers used in this study as a genomic resource for future GWAS and genomic-assisted breeding studies.

**Comment**. Line 114: Add the preposition “of” “Detailed characterization of…

**Response:** The sentence has been revised accordingly.

**Comment.** The introduction needs the inclusion of study hypothesis.

**Response:** A clear study hypothesis was added to the Introduction.

**Comment.** Clarify how the 93 lines were selected

**Response**: The 93 inbred lines were not selected based on prior performance evaluation but represent a set of lines with diverse genetic backgrounds developed through successive selfing.

**Comment**. Line 146–147: Use filtered for PIC or MAF. This is redundancy.

**Response:** The manuscript has been revised by removing PIC from the filtering criteria and retaining MAF as the primary filtering parameter. In addition, “Row” has been corrected to “Raw.”

**Comment.** The rationale for filtering SNPs with heterozygosity < 0.02 is unclear…

**Response:** The manuscript now clearly states that markers with high heterozygosity (>0.02), not low heterozygosity, were removed.

**Comment.** Missing data threshold of >30% may introduce noise.

**Response:** The threshold was retained to balance marker density and genome coverage in the GBS dataset. This justification has been clarified in the revised manuscript.

**Comment.** Do LD window size of 50 bp capture meaningful LD structure?

**Response:** We have addressed this issue earlier (Reviewer #3, Comment 1)

**Comment.** p < 0.1 versus p < 0.01 inconsistency

**Response:** The manuscript was revised to consistently define LD using the threshold r² ≥ 0.20.

**Comment**. The authors did not make clear that unmapped markers involved in LD analysis?

**Response:** These markers were excluded from LD analysis, as estimation of LD decay requires physical distances between markers. This clarification has now been added to the Methods section.

**Comment.** Increase membership probability threshold and STRUCTURE iterations.

**Response:** This comment was addressed earlier (Reviewer #3 and Reviewer #4). A membership coefficient threshold of Q ≥ 0.6 was retained. The burn-in period of 10,000 iterations and 50,000 MCMC repetitions provided stable and reproducible clustering results across independent runs.

**Comment**. Font size issue in table title**.**

**Response:** The table title font size has been corrected.

**Comment.** Across all pairwise SNPs, a mean of 0.50 is unrealistically high in maize.

**Response.** After re-evaluating the LD calculations, we have corrected the manuscript to report the genome-wide mean r² (0.067), which is consistent with expected values for maize. The revised results and corresponding interpretations have been updated accordingly.

**Comment.** *F*_ST_ = 0.05 is not entirely low.

**Response:** The interpretation was revised to “low to moderate genetic differentiation.

**Comment.** “Population differentiation” heading is unnecessary

**Response:** We agree that AMOVA, PhiPT, and pairwise FST are derived from the same analytical framework. Accordingly, the separate “Population Differentiation” heading has been removed

**Reviewer #5 Track change comments**

**Comment.** This sentence is needs to be revised.

**Response:** The sentence has been revised for clarity and readability

**Comment.** Please provide figure for Ethiopia productivity gap.

**Response:** The maize productivity gap between Ethiopia and the global average has now been included in the revised manuscript

**Comment.** Confusion between four populations and three clusters.

**Response:** The manuscript now consistently distinguishes predefined germplasm source groups from genetically inferred clusters.

**Comment.** But you are not doing marker-trait association analysis in this study

**Response:** Marker–trait association analysis was not conducted in this study. The statement in the abstract has been revised to clarify that the SNP dataset represents a resource for future studies.

**Comment.** MAF cannot exceed 0.5.

**Response:** The previously reported MAF value of was a typographical error and has been corrected.

**Comment**. It is good to present the results of population structure before diversity

**Response:** Presenting population structure results earlier can improve the logical flow. However, in this study, diversity within populations refers to predefined germplasm source groups, not the clusters inferred from population structure analysis. We have clarified this distinction in the manuscript and maintained the original organization.

**Comment**. It is good to provide the number in each population for better interpretation of the parameters

**Response**; thank you for this important observation. We have provided the number in each germplasm source groups.

**Comment**. Why didn’t you analyze all SNP pairs?

**Response:** All pairwise SNP comparisons were included in the revised LD analysis. The LD calculations and corresponding interpretations have been comprehensively revised throughout the manuscript.

**Comment**. Please cross-check this figure. Isn't 6.6%?

**Response:** This error is corrected in the later LD analysis and all the points raised regarding LD is corrected throughout the manuscript.

**Comment.** Is this referring to the number of pairs analyzed?

**Response:** This issue is also corrected in the new analysis (Reviewer #3, Comment 1)

**Comment**. Shouldn't these be part of discussion of results?

**Response;** We have moved LD discussion statements from result section to discussion section.

**Comment.** Which populations are you referring to?

**Response;** We clearly defined the study material to germplasm source group. The terminology is corrected throughout the manuscript.

**Comment**. The populations/sub-populations need to be clearly defined

**Response;** We clearly defined the study material to germplasm source group. The terminology is corrected throughout the manuscript.

**Comment.** Does it mean Cluster 2 had only 1 genotype?

**Response:** Cluster sizes were corrected to 40, 13, and 38 genotypes for Clusters I–III, respectively, with two outlier genotypes.

**Comment**. I think this would be better suited for discussion section

**Response;** We have moved the section to discussion section.

**Comment**. “Substantial diversity” is vague.

**Response:** The terminology was revised to “moderate genetic diversity” and supported using quantitative diversity estimates.

**Comment**. Your results do not show high gene diversity.

**Response:** We agree that the observed gene diversity is moderate rather than high. The manuscript has been revised accordingly, and terms such as “high genetic diversity” and “broad genetic base” have been replaced with more accurate descriptions.

**Comment**. It would be nice to bring more information about these populations.

**Response:** We have revised the manuscript to improve clarity by consistently using the term “germplasm source group” instead of “population” throughout the text. In addition, we have included a brief description of the composition of each group when discussing diversity patterns.

**Comment.** Ayesiga et al? Sserumaga et al? Please cross-check

**Response:** We have cross-checked the reference accordingly.

**Comment.** Which *F*_ST_ values are you referring to? Please be clear

**Response:** We have corrected the typing error.

**Comment**. Are you referring to this particular maize panel?

**Response:** The statement has been revised to clarify that PIC values reflect marker informativeness within the present panel rather than implying greater genetic diversity

**Comment.** But in line 296 you indicated a high gene diversity

**Response:** The manuscript now clearly states that the observed genetic diversity is moderate rather than high, and all overstatements have been corrected.

**Comment.** There appears to be contradiction in this statement.

**Response:** We have corrected the contradictory statements.

**Comment**. Are clusters the same as the populations you have been making references to

**Response;** Thank you for this important observation. We agree that the terminology and interpretation required clarification

**Comment.** How did you measure cluster distinctiveness?

**Response:** Cluster distinctiveness was assessed using STRUCTURE, PCA, neighbor-joining analysis, AMOVA, and *F*_ST_ estimates. However, the low *F*_ST_ values indicate weak differentiation among groups despite identifiable clustering.
