# Supplementary material for: High-density single nucleotide polymorphism markers analysis reveals the genetic diversity and population structure in tropical highland maize (Zea mays L.) inbred lines
Source: PLoS One. 2026 Jun 22;21(6):e0351845. doi: 10.1371/journal.pone.0351845 (PMC13286140; doi:10.1371/journal.pone.0351845)
Supplement: Peer review history

# Peer review: Decision Letter 0

**DOI:** 10.1371/journal.pone.0351845.r001

**Authors:** Dragan Perovic

19 Mar 2026

PONE-D-25-63384 High-density Single Nucleotide Polymorphism markers analysis reveals the genetic diversity and population structure in tropical highland maize (Zea mays L.) inbred lines PLOS One

Dear Dr. Gebre,

Thank you for submitting your manuscript to PLOS ONE. After careful consideration, we feel that it has merit but does not fully meet PLOS ONE’s publication criteria as it currently stands. Therefore, we invite you to submit a revised version of the manuscript that addresses the points raised during the review process.

Please submit your revised manuscript by May 03 2026 11:59PM. If you will need more time than this to complete your revisions, please reply to this message or contact the journal office at plosone@plos.org. When you're ready to submit your revision, log on to https://www.editorialmanager.com/pone/ and select the 'Submissions Needing Revision' folder to locate your manuscript file.

Please include the following items when submitting your revised manuscript:

- A letter that responds to each point raised by the academic editor and reviewer(s). You should upload this letter as a separate file labeled 'Response to Reviewers'.
- A marked-up copy of your manuscript that highlights changes made to the original version. You should upload this as a separate file labeled 'Revised Manuscript with Track Changes'.
- An unmarked version of your revised paper without tracked changes. You should upload this as a separate file labeled 'Manuscript'.

If you would like to make changes to your financial disclosure, please include your updated statement in your cover letter. Guidelines for resubmitting your figure files are available below the reviewer comments at the end of this letter.

If applicable, we recommend that you deposit your laboratory protocols in protocols.io to enhance the reproducibility of your results. Protocols.io assigns your protocol its own identifier (DOI) so that it can be cited independently in the future. For instructions see: https://journals.plos.org/plosone/s/submission-guidelines#loc-laboratory-protocols. Additionally, PLOS ONE offers an option for publishing peer-reviewed Lab Protocol articles, which describe protocols hosted on protocols.io. Read more information on sharing protocols at https://plos.org/protocols?utm_medium=editorial-email&utm_source=authorletters&utm_campaign=protocols.

We look forward to receiving your revised manuscript.

Kind regards,

Muhammad Abdul Rehman Rashid, PhD

Academic Editor

PLOS One

Journal Requirements:

When submitting your revision, we need you to address these additional requirements.

1. Please ensure that your manuscript meets PLOS ONE's style requirements, including those for file naming. The PLOS ONE style templates can be found at

https://journals.plos.org/plosone/s/file?id=wjVg/PLOSOne_formatting_sample_main_body.pdf and https://journals.plos.org/plosone/s/file?id=ba62/PLOSOne_formatting_sample_title_authors_affiliations.pdf

2. Please note that PLOS One has specific guidelines on code sharing for submissions in which author-generated code underpins the findings in the manuscript. In these cases, we expect all author-generated code to be made available without restrictions upon publication of the work. Please review our guidelines at https://journals.plos.org/plosone/s/materials-and-software-sharing#loc-sharing-code and ensure that your code is shared in a way that follows best practice and facilitates reproducibility and reuse.

3. We note that you have indicated that there are restrictions to data sharing for this study. PLOS only allows data to be available upon request if there are legal or ethical restrictions on sharing data publicly. For more information on unacceptable data access restrictions, please see http://journals.plos.org/plosone/s/data-availability#loc-unacceptable-data-access-restrictions.

Before we proceed with your manuscript, please address the following prompts:

a) If there are ethical or legal restrictions on sharing a de-identified data set, please explain them in detail (e.g., data contain potentially identifying or sensitive patient information, data are owned by a third-party organization, etc.) and who has imposed them (e.g., a Research Ethics Committee or Institutional Review Board, etc.). Please also provide contact information for a data access committee, ethics committee, or other institutional body to which data requests may be sent.

b) If there are no restrictions, please upload the minimal anonymized data set necessary to replicate your study findings to a stable, public repository and provide us with the relevant URLs, DOIs, or accession numbers. For a list of recommended repositories, please see

https://journals.plos.org/plosone/s/recommended-repositories. You also have the option of uploading the data as Supporting Information files, but we would recommend depositing data directly to a data repository if possible.

We will update your Data Availability statement on your behalf to reflect the information you provide.

4. In the online submission form, you indicated that the data will be available upon request from corresponding author

All PLOS journals now require all data underlying the findings described in their manuscript to be freely available to other researchers, either 1. In a public repository, 2. Within the manuscript itself, or 3. Uploaded as supplementary information.

This policy applies to all data except where public deposition would breach compliance with the protocol approved by your research ethics board. If your data cannot be made publicly available for ethical or legal reasons (e.g., public availability would compromise patient privacy), please explain your reasons on resubmission and your exemption request will be escalated for approval.

5. Please amend your authorship list in your manuscript file to include author worknesh Terefe Gebre, Demissew Ababulgu, Tilahun Negassa, Tileye Senbeta.

6. Please amend the manuscript submission data (via Edit Submission) to include author Worknesh Terefe, Demissew Abakemal, Tilahun Mekonnen, Tileye Feyissa.

7. Please include captions for your Supporting Information files at the end of your manuscript, and update any in-text citations to match accordingly. Please see our Supporting Information guidelines for more information: http://journals.plos.org/plosone/s/supporting-information.

8. We note that there is identifying data in the Supporting Information file <Supplementary tables.docx >. Due to the inclusion of these potentially identifying data, we have removed this file from your file inventory. Prior to sharing human research participant data, authors should consult with an ethics committee to ensure data are shared in accordance with participant consent and all applicable local laws.

Data sharing should never compromise participant privacy. It is therefore not appropriate to publicly share personally identifiable data on human research participants. The following are examples of data that should not be shared:

-Name, initials, physical address

-Ages more specific than whole numbers

-Internet protocol (IP) address

-Specific dates (birth dates, death dates, examination dates, etc.)

-Contact information such as phone number or email address

-Location data

-ID numbers that seem specific (long numbers, include initials, titled “Hospital ID”) rather than random (small numbers in numerical order)

Data that are not directly identifying may also be inappropriate to share, as in combination they can become identifying. For example, data collected from a small group of participants, vulnerable populations, or private groups should not be shared if they involve indirect identifiers (such as sex, ethnicity, location, etc.) that may risk the identification of study participants.

Additional guidance on preparing raw data for publication can be found in our Data Policy (https://journals.plos.org/plosone/s/data-availability#loc-human-research-participant-data-and-other-sensitive-data) and in the following article: http://www.bmj.com/content/340/bmj.c181.long.

Please remove or anonymize all personal information (<specific identifying information in file to be removed>), ensure that the data shared are in accordance with participant consent, and re-upload a fully anonymized data set. Please note that spreadsheet columns with personal information must be removed and not hidden as all hidden columns will appear in the published file.

9. If the reviewer comments include a recommendation to cite specific previously published works, please review and evaluate these publications to determine whether they are relevant and should be cited. There is no requirement to cite these works unless the editor has indicated otherwise.

10. Please review your reference list to ensure that it is complete and correct. If you have cited papers that have been retracted, please include the rationale for doing so in the manuscript text, or remove these references and replace them with relevant current references. Any changes to the reference list should be mentioned in the rebuttal letter that accompanies your revised manuscript. If you need to cite a retracted article, indicate the article’s retracted status in the References list and also include a citation and full reference for the retraction notice.

[Note: HTML markup is below. Please do not edit.]

Reviewers' comments:

Reviewer's Responses to Questions

**Comments to the Author**

1. Is the manuscript technically sound, and do the data support the conclusions?

The manuscript must describe a technically sound piece of scientific research with data that supports the conclusions. Experiments must have been conducted rigorously, with appropriate controls, replication, and sample sizes. The conclusions must be drawn appropriately based on the data presented.

Reviewer #1: Yes

Reviewer #2: Yes

Reviewer #3: Yes

Reviewer #4: Partly

Reviewer #5: Yes

**********

2. Has the statistical analysis been performed appropriately and rigorously?

Reviewer #1: Yes

Reviewer #2: Yes

Reviewer #3: Yes

Reviewer #4: No

Reviewer #5: Yes

**********

3. Have the authors made all data underlying the findings in their manuscript fully available?

The PLOS Data policy requires authors to make all data underlying the findings described in their manuscript fully available without restriction, with rare exception (please refer to the Data Availability Statement in the manuscript PDF file). The data should be provided as part of the manuscript or its supporting information, or deposited to a public repository. For example, in addition to summary statistics, the data points behind means, medians and variance measures should be available. If there are restrictions on publicly sharing data—e.g. participant privacy or use of data from a third party—those must be specified.

Reviewer #1: Yes

Reviewer #2: Yes

Reviewer #3: No

Reviewer #4: No

Reviewer #5: Yes

**********

4. Is the manuscript presented in an intelligible fashion and written in standard English?

PLOS ONE does not copyedit accepted manuscripts, so the language in submitted articles must be clear, correct, and unambiguous. Any typographical or grammatical errors should be corrected at revision, so please note any specific errors here.

Reviewer #1: Yes

Reviewer #2: Yes

Reviewer #3: Yes

Reviewer #4: No

Reviewer #5: Yes

**********

5. Review Comments to the Author

Please use the space provided to explain your answers to the questions above. You may also include additional comments for the author, including concerns about dual publication, research ethics, or publication ethics. (Please upload your review as an attachment if it exceeds 20,000 characters)

Reviewer #1: Lines 127-129 can be elaborated for development of the inbred lines mentioning the hybridization sources and selfed generations.

Lines 131-132 needs elaboration as to what adaptation and disease resistance strategies were adopted for evaluation of inbred lines

Reviewer #2: The work is fundamental and presentation is fine. but authors should give clear high resolution figures. the abstrct shouldbe more informative.If possible the gst and rst value may be added.authors should see if the SNPS can be associated with specific trait. It may be included in discussion.

Reviewer #3: This study presents a comprehensive genetic analysis of 93 tropical highland maize inbred lines using high-density SNP markers. The authors assess genetic diversity, population structure, and linkage disequilibrium, offering valuable insights for maize breeding programs in East African highlands. The manuscript is well-structured and addresses an important topic in crop improvement. However, authors

The authors state that data will be available upon request. However there are major concerns:

1. The LD decay analysis mentions a window size of 50 bp—this seems unusually small for maize. Please confirm and clarify whether this refers to a sliding-window step or to something else.

2. The STRUCTURE analysis used K = 1–10 with 10,000 burn-in and 50,000 MCMC iterations. These are acceptable but relatively low for complex datasets. Consider justifying or noting this as a limitation.

3. The PCA explains only ~8.8% of the total variation (PC1 + PC2). While this is common in highly diverse panels, it may be worth discussing the limitations of PCA in capturing complex structure and the value of complementary methods like STRUCTURE and NJ trees.

4. Table 3 shows chromosome 6 with 0 significant LD pairs—this is unusual and may warrant a check or explanation (e.g., low marker density, filtering artifacts).

5. Please discuss why population 4 might have lower diversity. Could it be due to selection history or genetic drift? What are the practical implications of the LD decay rate for GWAS resolution in this panel? Additionally, consider more explicitly comparing your findings with those from other highland maize studies in Africa or similar environments.

6. Per journal requirement, authors must deposit the SNP data in a public repository (e.g., Figshare, Dryad, or a genomic database) and provide the accession number in the manuscript.

Additionally, some typos must be corrected: Figures are generally clear, but ensure all axes are labeled with units (e.g., Fig 4: Distance in bp). Table 3: The column header "Pr²" is unclear: should it be "Mean r²"? Table S2 (Nm values) is mentioned but not fully described in the text; ensure that all supplementary files are referenced and explained.

In Abstract: The sentence beginning "11,203 single nucleotide polymorphism (SNP) markers genotyped..." is missing a verb (e.g., "were used"). Revise for clarity.

Keywords: Consider adding "GWAS" or "Genomic selection" if relevant to future applications.

Line 185: (additional file1) must be (Additional File S1), and accordingly, the Supporting Information section must be corrected too.

Line 256: "Cluster 1 contained the largest germplasm (54 lines, 58%)" – rephrase to "largest number of lines" or "most lines".

Line 283: "The assessment of genetic diversity..." – this sentence is a general statement; consider moving to the Introduction or shortening.

Technical Corrections

Check for inconsistent spacing (e.g., "p< 0.01" should be "p < 0.01").

Use italic formatting for p-values and FST consistently.

Table 5: The diagonal values (0.001, 0.001, 0.009) are listed under "Sub-populations 1" column, this may confuse readers. Use a standard format with FST above diagonal and genetic distance below.

Reviewer #4: This manuscript presents a comprehensive SNP-based analysis of genetic diversity, population structure, and linkage disequilibrium in tropical highland maize inbred lines. The topic is scientifically relevant and important for maize breeding programs, especially in sub-Saharan Africa. The dataset is substantial, and the analytical approaches are appropriate. However, the manuscript requires major revision to meet PLOS ONE publication standards, particularly in language quality, methodological clarity, data availability, and interpretation of results.

Major Strengths

Scientific relevance:

The study addresses a significant knowledge gap in the genetic characterization of tropical highland maize germplasm, which is important for breeding, conservation, and climate-resilient crop development.

High-quality dataset:

The use of over 11,000 high-quality SNP markers across 93 inbred lines provides robust genomic resolution for population genetic analysis.

Comprehensive analytical framework:

The authors employ multiple complementary analyses, including genetic diversity indices, STRUCTURE clustering, PCA, phylogenetic analysis, AMOVA, and LD decay analysis, which together strengthen the study.

Breeding implications:

The manuscript provides potentially valuable insights for heterotic grouping and parental selection in maize hybrid development.

Major Concerns and Required Revisions

1. Language and Writing Quality (Major Issue)

The manuscript contains numerous grammatical, typographical, and syntactic errors that substantially impair readability and clarity. Professional English language editing is strongly recommended. Several sentences are poorly constructed, repetitive, or ambiguous.

2. Methodological Clarity and Justification

SNP filtering thresholds:

The authors applied several filtering criteria (PIC < 0.1, MAF < 0.05, heterozygosity < 0.02, missing data > 30%), but no justification or citation is provided. These thresholds should be supported with references or methodological rationale.

LD window size:

The manuscript reports an LD window size of 50 bp, which is biologically unrealistic for maize population studies. This likely reflects either a typographical or analytical error. The authors should clarify the actual window size used and revise accordingly.

STRUCTURE parameters:

The burn-in and MCMC iterations appear relatively low for population genetic inference. Justification for these choices should be provided, or additional runs should be performed to ensure robust inference.

3. Interpretation of Linkage Disequilibrium Results

The reported LD decay (~3.33 Mb) is considerably slower than expected for maize and is inconsistent with many previous studies. This may reflect population stratification, recent selection bottlenecks, or marker filtering bias. The authors should compare their findings with published maize LD studies and provide a biological explanation for the unusually slow decay.

4. Population Structure Interpretation

Although three genetic clusters are identified, the biological and breeding significance of these clusters is not adequately discussed. The authors should strengthen interpretation by linking population structure results to pedigree background, geographic origin, adaptation patterns, and implications for heterotic grouping.

5. FST Inconsistencies

The manuscript reports contradictory FST values in different sections. While the text mentions values as high as 0.716, the tables show FST values below 0.01. This discrepancy must be corrected, and population differentiation should be interpreted accordingly.

6. Data Availability (Mandatory Revision)

The current Data Availability Statement does not comply with PLOS ONE policy. All genotype datasets and supporting data must be deposited in a publicly accessible repository, and accession numbers or DOIs must be provided.

Minor Issues:

Several figures require improved resolution and clearer legends.

Some tables contain formatting inconsistencies.

Citations should be updated and formatted consistently.

Reviewer #5: The manuscript tilted “High-density Single Nucleotide Polymorphism markers analysis reveals the genetic diversity and population structure in tropical highland maize (Zea mays L.) Inbred lines” is well written scientifically and analytically. Also, it contributes to the understanding of tropical highland maize germplasm for improvement. The manuscript could benefit more by using some of the comments in the attachment

**********

6. PLOS authors have the option to publish the peer review history of their article (what does this mean?). If published, this will include your full peer review and any attached files.

If you choose “no”, your identity will remain anonymous but your review may still be made public.

**Do you want your identity to be public for this peer review?** For information about this choice, including consent withdrawal, please see our Privacy Policy.

Reviewer #1: No

Reviewer #2: No

Reviewer #3: **Yes:** Ibrokhim Y. Abdurakhmonov

Reviewer #4: No

Reviewer #5: **Yes:** Idris Ishola Adejumobi (PhD)

**********

[NOTE: If reviewer comments were submitted as an attachment file, they will be attached to this email and accessible via the submission site. Please log into your account, locate the manuscript record, and check for the action link "View Attachments". If this link does not appear, there are no attachment files.]

To ensure your figures meet our technical requirements, please review our figure guidelines: https://journals.plos.org/plosone/s/figures

You may also use PLOS’s free figure tool, NAAS, to help you prepare publication quality figures: https://journals.plos.org/plosone/s/figures#loc-tools-for-figure-preparation.

NAAS will assess whether your figures meet our technical requirements by comparing each figure against our figure specifications.

Attachment Submitted filename: ManuscriptPLOSone Review.doc

Attachment Submitted filename: PONE REVIEW.docx

# Author comment: Author response to Decision Letter 1

**DOI:** 10.1371/journal.pone.0351845.r002

25 May 2026

Response to reviewers

We sincerely thank the Editor and Reviewers for their careful evaluation and constructive comments. Their suggestions substantially improved the clarity, methodological rigor, and overall quality of the manuscript. All revisions in the manuscript are highlighted in bold text.

We have carefully revised the manuscript in response to all comments. Major revisions include:

• Reanalysis and correction of linkage disequilibrium (LD) results and interpretation,

• Improvement of methodological descriptions and SNP filtering criteria,

• Revision of population structure and genetic diversity interpretations,

• Correction of inconsistencies in FST reporting,

• Improvement of figure quality, table formatting, and supplementary information,

• Clarification of terminology distinguishing predefined germplasm source groups from genetically inferred clusters,

• Revision of language, grammar, and scientific clarity throughout the manuscript, and

• Deposition of all datasets in the Dryad Digital Repository in accordance with PLOS ONE data availability requirements.

In addition, all statistical analyses, particularly LD estimation and population differentiation analyses, were carefully re-evaluated and verified to ensure consistency and accuracy throughout the manuscript.

Reviewer #1

Comment 1. Lines 127-129 can be elaborated for development of the inbred lines

Response: Additional details were added describing the origin and development of the inbred lines.

Comment 2. Clarify adaptation and disease resistance strategies.

Response: The manuscript now clarifies that adaptation and disease resistance strategies during line development.

Reviewer #2

Comment 1. Provide high-resolution figures.

Response: All figures were replaced with high-resolution versions (≥ 300 dpi), and figure legends were revised for improved clearly and readability.

Comment 2. The abstract should be more informative.

Response: The abstract was revised to improve clarity and informativeness by correcting LD statistics, refining diversity descriptions, and revising the concluding statements.

Comment 3. If possible, the Gst and Rst value may be added.

Response: Because the present study used biallelic SNP markers, AMOVA and FST based approaches were considered more appropriate and are widely applied in SNP based population genetic studies. Therefore, the manuscript retains the AMOVA-based estimates of genetic differentiation.

Comment 4. Authors should see if the SNPs can be associated with specific traits.

Response: GWAS framework was beyond the scope of the present study. Nevertheless, the discussion now emphasizes that the SNP dataset provides a valuable resource for future marker-trait association studies and genomic-assisted breeding.

Reviewer #3

Comment 1. The LD decay analysis mentions a window size of 50 bp-this seems unusual

Response: The previously reported “50 bp” value resulted from misinterpretation of the TASSEL parameter. In TASSEL, the LD window refers to the number of adjacent markers rather than physical distance. LD was recalculated using physically mapped SNPs, LOESS smoothing, and 10 kb distance bins. The revised analysis estimated genome-wide LD decay at approximately 93.82 kb (r² = 0.2), consistent with previous maize studies. All related sections, figures, and tables were updated accordingly

Comment 2. The STRUCTURE analysis used relatively low burn-in and MCMC iterations.

Response: The STRUCTURE analysis used 10,000 burn-in iterations and 50,000 MCMC repetitions. Multiple independent runs produced stable clustering patterns consistently supported by STRUCTURE, PCA, and neighbor-joining analyses. This justification has been clarified in the manuscript.

Comment 3. The PCA explains only ~8.8% of the total variation.

Response: The Discussion section has been revised to clarify that PCA alone may have limited power to resolve complex population structure, and therefore complementary analyses including STRUCTURE and neighbor-joining clustering were used to provide a more comprehensive assessment.

Comment 4. Table 3 shows chromosome 6 with 0 significant LD pairs.

Response: This resulted from a reporting error. After correction, chromosome 6 contained 3,027 significant LD pairs (r² ≥ 0.2). Table 3 and related text were updated.

Comment 5. Discuss low diversity in population 4 and the implications of LD decay for GWAS.

Response: The Discussion was expanded to explain that the lower diversity observed in germplasm source group 4 is likely associated with selection history and genetic drift, which may have reduced allelic variation through recurrent breeding and shared ancestry. The implications of the observed LD decay (~93.82 kb) for GWAS resolution were also discussed.

Comment 6. Authors must deposit the SNP data in a public repository

Response: The Data Availability Statement does not comply with PLOS ONE policy.

Response: All genotype datasets and supporting files were deposited in the Dryad Digital Repository and are publicly accessible at: https://doi.org/10.5061/dryad.2547d7x6d

Minor Comments

Response: All figures have been revised to ensure clear axis labeling and inclusion of appropriate units. Table headings and supplementary materials were corrected and standardized throughout the manuscript. Formatting inconsistencies, spacing, statistical notation, and terminology were also revised for consistency.

Comment. In Abstract: The sentence missing a verb should be clarified.

Response: The sentence has been revised to include the appropriate verb for clarity.

Comment. Consider adding "GWAS" or "Genomic selection" if relevant to future applications.

Response: The keywords section has been revised to include GWAS.

Comment. Additional File S1, and the Supporting Information section must be corrected too.

Response: The formatting has been corrected to “Additional File S1,” and the Supporting Information section has been revised accordingly.

Comment. "Cluster 1 contained the largest germplasm (54 lines, 58%)" – rephrase it

Response: The sentence has been revised and improved.

Comment. The sentence is a general statement; consider moving to the Introduction

Response: The sentence has been shortened to improve conciseness while maintaining its relevance in the Discussion section.

Comment. Check for inconsistent spacing and consistent italic formatting for statistical symbols.

Response: We have corrected the spacing and standardized the formatting of statistical symbols (p, FST, and r²) throughout the manuscript.

Comment. Table 5 formatting confusion

Response: Table 5 was reformatted to improve clarity and consistency of pairwise FST presentation.

Reviewer #4

Comment. The manuscript contains numerous grammatical, typographical, and syntactic errors.

Response: The manuscript was thoroughly revised for grammar, syntax, and readability. Repetitive and ambiguous statements were corrected, and the manuscript underwent comprehensive English language editing.

Comment. SNP filtering criteria lack justification.

Response. The manuscript now provides justification and supporting references for SNP filtering criteria. Markers with low MAF, excessive missing data, and high heterozygosity (> 0.02) were removed to improve marker quality and analytical robustness.

Comment. The LD window size of 50 bp is unrealistic.

Response: This issue was addressed earlier (Reviewer #3, Comment 1). Briefly, the “50 bp” value resulted from misinterpretation of the TASSEL parameter; the analysis used a window of 50 adjacent markers rather than physical distance.

Comment. STRUCTURE iterations appear low.

Response: This issue was addressed earlier (Reviewer #3, Comment 2). The selected burn-in and MCMC values produced stable and reproducible clustering patterns across independent runs. This clarification was added to the manuscript.

Comment. The reported LD decay (~3.33 Mb) is inconsistent with maize studies.

Response: The LD analysis was re-evaluated using only physically mapped SNPs. The corrected genome-wide LD decay estimate was approximately 93.82 kb (r2 = 0.2), consistent with previous maize studies. All related analyses, figure, and interpretations were revised accordingly.

Comment. The biological and breeding significance of clusters is not adequately discussed.

Response: The Discussion was expanded to relate the identified clusters to pedigree background, germplasm origin, adaptation, and breeding history, as well as their implications for maize improvement and heterotic grouping.

Comment. The manuscript reports contradictory FST values in different sections.

Response: We carefully rechecked the analysis. Pairwise FST values ranged from 0.001 to 0.009, indicating low to moderate differentiation.

Comment. The Data Availability Statement does not comply with PLOS ONE policy.

Response: This was addressed earlier (Reviewer #3, Comment 6).

Comment. Improve figure quality legends.

Response: All figures were improved and was addressed earlier (Reviewer #2, Comment 1)

Comment. Some tables contain formatting inconsistencies.

Response: All tables were reformatted for consistency in style, spacing, alignment, and notation.

Comment. Citations should be updated and formatted consistently.

Response: All citations and references were reviewed and standardized according to journal guidelines using EndNote.

Reviewer #5

Comment 1. Clarify the mean r2 of 0.50

Response: The revised manuscript now reports a genome-wide mean r2 value of 0.067 across all pairwise SNP comparisons. This was addressed earlier (Reviewer #3, Comment 1).

Comment 2. Lines 59-62: furthermore, the identified SNP…what SNPs were identified?

Response: The statement was revised for clarify. The text now refers to the SNP markers used in this study as a genomic resource for future GWAS and genomic-assisted breeding studies.

Comment. Line 114: Add the preposition “of” “Detailed characterization of…

Response: The sentence has been revised accordingly.

Comment. The introduction needs the inclusion of study hypothesis.

Response: A clear study hypothesis was added to the Introduction.

Comment. Clarify how the 93 lines were selected

Response: The 93 inbred lines were not selected based on prior performance evaluation but represent a set of lines with diverse genetic backgrounds developed through successive selfing.

Comment. Line 146–147: Use filtered for PIC or MAF. This is redundancy.

Response: The manuscript has been revised by removing PIC from the filtering criteria and retaining MAF as the primary filtering parameter. In addition, “Row” has been corrected to “Raw.”

Comment. The rationale for filtering SNPs with heterozygosity < 0.02 is unclear…

Response: The manuscript now clearly states that markers with high heterozygosity (>0.02), not low heterozygosity, were removed.

Comment. Missing data threshold of >30% may introduce noise.

Response: The threshold was retained to balance marker density and genome coverage in the GBS dataset. This justification has been clarified in the revised manuscript.

Comment. Do LD window size of 50 bp capture meaningful LD structure?

Response: We have addressed this issue earlier (Reviewer #3, Comment 1)

Comment. p < 0.1 versus p < 0.01 inconsistency

Response: The manuscript was revised to consistently define LD using the threshold r² ≥ 0.20.

Comment. The authors did not make clear that unmapped markers involved in LD analysis?

Response: These markers were excluded from LD analysis, as estimation of LD decay requires physical distances between markers. This clarification has now been added to the Methods section.

Comment. Increase membership probability threshold and STRUCTURE iterations.

Response: This comment was addressed earlier (Reviewer #3 and Reviewer #4). A membership coefficient threshold of Q ≥ 0.6 was retained. The burn-in period of 10,000 iterations and 50,000 MCMC repetitions provided stable and reproducible clustering results across independent runs.

Comment. Font size issue in table title.

Response: The table title font size has been corrected.

Comment. Across all pairwise SNPs, a mean of 0.50 is unrealistically high in maize.

Response. After re-evaluating the LD calculations, we have corrected the manuscript to report the genome-wide mean r² (0.067), which is consistent with expected values for maize. The revised results and corresponding interpretations have been updated accordingly.

Comment. FST = 0.05 is not entirely low.

Response: The interpretation was revised to “low to moderate genetic differentiation.

Comment. “Population differentiation” heading is unnecessary

Response: We agree that AMOVA, PhiPT, and pairwise FST are derived from the same analytical framework. Accordingly, the separate “Population Differentiation” heading has been removed

Reviewer #5 Track change comments

Comment. This sentence is needs to be revised.

Response: The sentence has been revised for clarity and readability

Comment. Please provide figure for Ethiopia productivity gap.

Response: The maize productivity gap between Ethiopia and the global average has now been included in the revised manuscript

Comment. Confusion between four populations and three clusters.

Response: The manuscript now consistently distinguishes predefined germplasm source groups from genetically inferred clusters.

Comment. But you are not doing marker-trait association analysis in this study

Response: Marker–trait association analysis was not conducted in this study. The statement in the abstract has been revised to clarify that the SNP dataset represents a resource for future studies.

Comment. MAF cannot exceed 0.5.

Response: The previously reported MAF value of was a typographical error and has been corrected.

Comment. It is good to present the results of population structure before diversity

Response: Presenting population structure results earlier can improve the logical flow. However, in this study, diversity within populations refers to predefined germplasm source groups, not the clusters inferred from population structure analysis. We have clarified this distinction in the manuscript and maintained the original organization.

Comment. It is good to provide the number in each population for better interpretation of the parameters

Response; thank you for this important observation. We have provided the number in each germplasm source groups.

Comment. Why didn’t you analyze all SNP pairs?

Response: All pairwise SNP comparisons were included in the revised LD analysis. The LD calculations and corresponding interpretations have been comprehensively revised throughout the manuscript.

Comment. Please cross-check this figure. Isn't 6.6%?

Response: This error is corrected in the later LD analysis and all the points raised regarding LD is corrected throughout the manuscript.

Comment. Is this referring to the number of pairs analyzed?

Response: This issue is also corrected in the new analysis (Reviewer #3, Comment 1)

Comment. Shouldn't these be part of discussion of results?

Response; We have moved LD discussion statements from result section to discussion section.

Comment. Which populations are you referring to?

Response; We clearly defined the study material to germplasm source group. The terminology is corrected throughout the manuscript.

Comment. The populations/sub-populations need to be clearly defined

Response; We clearly defined the study material to germplasm source group. The terminology is corrected throughout the manuscript.

Comment. Does it mean Cluster 2 had only 1 genotype?

Response: Cluster sizes were corrected to 40, 13, and 38 genotypes for Clusters I–III, respectively, with two outlier genotypes.

Comment. I think this would be better suited for discussion section

Response; We have moved the section to discussion section.

Comment. “Substantial diversity” is vague.

Response: The terminology was revised to “moderate genetic diversity” and supported using quantitative diversity estimates.

Comment. Your results do not show high gene diversity.

Response: We agree that the observed gene diversity is moderate rather than high. The manuscript has been revised accordingly, and terms such as “high genetic diversity” and “broad genetic base” have been replaced with more accurate descriptio

Attachment Submitted filename: Response to Reviewers.docx

# Editor report: Decision Letter 1

**DOI:** 10.1371/journal.pone.0351845.r003

**Authors:** Dragan Perovic, Dragan Perovic

2 Jun 2026

<p>High-density Single Nucleotide Polymorphism markers analysis reveals the genetic diversity and population structure in tropical highland maize (Zea mays L.) inbred lines

PONE-D-25-63384R1

Dear Dr. Gebre,

We’re pleased to inform you that your manuscript has been judged scientifically suitable for publication and will be formally accepted for publication once it meets all outstanding technical requirements.

Within one week, you’ll receive an e-mail detailing the required amendments. When these have been addressed, you’ll receive a formal acceptance letter and your manuscript will be scheduled for publication.

An invoice will be generated when your article is formally accepted. Please note, if your institution has a publishing partnership with PLOS and your article meets the relevant criteria, all or part of your publication costs will be covered. Please make sure your user information is up-to-date by logging into Editorial Manager at Editorial Manager® and clicking the ‘Update My Information' link at the top of the page. For questions related to billing, please contact billing support.

If your institution or institutions have a press office, please notify them about your upcoming paper to help maximize its impact. If they’ll be preparing press materials, please inform our press team as soon as possible -- no later than 48 hours after receiving the formal acceptance. Your manuscript will remain under strict press embargo until 2 pm Eastern Time on the date of publication. For more information, please contact onepress@plos.org.

Kind regards,

Dragan Perovic, Ph.D

Academic Editor

PLOS One

Additional Editor Comments (optional):

Reviewers' comments:

# Editor report: Acceptance letter

**DOI:** 10.1371/journal.pone.0351845.r004

**Authors:** Dragan Perovic, Dragan Perovic

PONE-D-25-63384R1

PLOS One

Dear Dr. Gebre,

I'm pleased to inform you that your manuscript has been deemed suitable for publication in PLOS One. Congratulations! Your manuscript is now being handed over to our production team.

At this stage, our production department will prepare your paper for publication. This includes ensuring the following:

* All references, tables, and figures are properly cited

* All relevant supporting information is included in the manuscript submission,

* There are no issues that prevent the paper from being properly typeset

You will receive further instructions from the production team, including instructions on how to review your proof when it is ready. Please keep in mind that we are working through a large volume of accepted articles, so please give us a few days to review your paper and let you know the next and final steps.

Lastly, if your institution or institutions have a press office, please let them know about your upcoming paper now to help maximize its impact. If they'll be preparing press materials, please inform our press team within the next 48 hours. Your manuscript will remain under strict press embargo until 2 pm Eastern Time on the date of publication. For more information, please contact onepress@plos.org.

You will receive an invoice from PLOS for your publication fee after your manuscript has reached the completed accept phase. If you receive an email requesting payment before acceptance or for any other service, this may be a phishing scheme. Learn how to identify phishing emails and protect your accounts at https://explore.plos.org/phishing.

If we can help with anything else, please email us at customercare@plos.org.

Thank you for submitting your work to PLOS ONE and supporting open access.

Kind regards,

PLOS ONE Editorial Office Staff

on behalf of

Dr. Dragan Perovic

Academic Editor

PLOS One
